# Integration of genomic, transcriptomic and proteomic data identifies two biologically distinct subtypes of invasive lobular breast cancer

**DOI:** 10.1038/srep18517

**Published:** 2016-01-05

**Authors:** Magali Michaut, Suet-Feung Chin, Ian Majewski, Tesa M. Severson, Tycho Bismeijer, Leanne de Koning, Justine K. Peeters, Philip C. Schouten, Oscar M. Rueda, Astrid J. Bosma, Finbarr Tarrant, Yue Fan, Beilei He, Zheng Xue, Lorenza Mittempergher, Roelof J.C. Kluin, Jeroen Heijmans, Mireille Snel, Bernard Pereira, Andreas Schlicker, Elena Provenzano, Hamid Raza Ali, Alexander Gaber, Gillian O’Hurley, Sophie Lehn, Jettie J.F. Muris, Jelle Wesseling, Elaine Kay, Stephen John Sammut, Helen A. Bardwell, Aurélie S. Barbet, Floriane Bard, Caroline Lecerf, Darran P. O’Connor, Daniël J. Vis, Cyril H. Benes, Ultan McDermott, Mathew J. Garnett, Iris M. Simon, Karin Jirström, Thierry Dubois, Sabine C. Linn, William M. Gallagher, Lodewyk F.A. Wessels, Carlos Caldas, Rene Bernards

**Affiliations:** 1 Division of Molecular Carcinogenesis, The Netherlands Cancer Institute, Plesmanlaan 121, 1066 CX Amsterdam, The Netherlands; 2 Cancer Research UK Cambridge Institute, University of Cambridge, Li Ka Shing Centre, Robinson Way, Cambridge, CB2 0RE, UK; 3 Division of Molecular Pathology, The Netherlands Cancer Institute, Plesmanlaan 121, 1066 CX Amsterdam, The Netherlands; 4 Translational Research Department, Institut Curie, 26 rue d’Ulm, 75248 Paris cedex 05, France; 5 Agendia NV, Science Park 406, 1098 XH Amsterdam, The Netherlands; 6 Cancer Biology and Therapeutics Laboratory, UCD School of Biomolecular and Biomedical Science, UCD Conway Institute, University College Dublin, Belfield, Dublin 4, Ireland; 7 OncoMark Limited, NovaUCD, Belfield Innovation Park, Dublin 4, Ireland; 8 Genomic Core Facility, The Netherlands Cancer Institute, Plesmanlaan 121, 1066 CX Amsterdam, The Netherlands; 9 Cambridge Experimental Cancer Medicine Centre (ECMR) and NIHR Cambridge Biomedical Research Centre, Cambridge University Hospitals NHS Foundation Trust, Cambridge CB2 0QQ, UK; 10 Cambridge Breast Unit and Cambridge University Hospitals, NHS Foundation Trust, Hills Road, Cambridge CB2 0QQ, UK; 11 Department of Pathology, University of Cambridge, Tennis Court Road, Cambridge, CB2 1QP, UK; 12 Department of Clinical Sciences Lund, Division of Oncology and Pathology, Lund University, SE-221 85 Lund, Sweden; 13 Department of Pathology, RCSI ERC, Beaumont Hospital, Dublin 9, Ireland; 14 Massachusetts General Hospital Cancer Center, Harvard Medical School, Charlestown, Massachusetts 02129, USA; 15 Cancer Genome Project, Wellcome Trust Sanger Institute, Hinxton CB10 1SA, UK; 16 Division of Medical Oncology, The Netherlands Cancer Institute, Plesmanlaan 121, 1066 CX Amsterdam, The Netherlands; 17 Department of Pathology, University Medical Center Utrecht, Heidelberglaan 100, 3584 CX Utrecht, The Netherlands; 18 Department of EEMCS, Delft University of Technology, Delft, The Netherlands; 19 Department of Oncology, University of Cambridge, Addenbrooke’s Hospital, Hills Road, Cambridge, CB2 0QQ, UK; 20 Cancer Genomics Netherlands, The Netherlands Cancer Institute, Plesmanlaan 121, 1066 CX Amsterdam, The Netherlands

## Abstract

Invasive lobular carcinoma (ILC) is the second most frequently occurring histological breast cancer subtype after invasive ductal carcinoma (IDC), accounting for around 10% of all breast cancers. The molecular processes that drive the development of ILC are still largely unknown. We have performed a comprehensive genomic, transcriptomic and proteomic analysis of a large ILC patient cohort and present here an integrated molecular portrait of ILC. Mutations in *CDH1* and in the PI3K pathway are the most frequent molecular alterations in ILC. We identified two main subtypes of ILCs: (i) an immune related subtype with mRNA up-regulation of *PD-L1, PD-1* and *CTLA-4* and greater sensitivity to DNA-damaging agents in representative cell line models; (ii) a hormone related subtype, associated with Epithelial to Mesenchymal Transition (EMT), and gain of chromosomes 1q and 8q and loss of chromosome 11q. Using the somatic mutation rate and eIF4B protein level, we identified three groups with different clinical outcomes, including a group with extremely good prognosis. We provide a comprehensive overview of the molecular alterations driving ILC and have explored links with therapy response. This molecular characterization may help to tailor treatment of ILC through the application of specific targeted, chemo- and/or immune-therapies.

Breast cancer is a heterogeneous disease and has traditionally been subdivided into distinct histological subtypes based on cell morphology. About 60–75% of breast cancers are invasive ductal carcinomas (IDC)^1^. The next most common subtype is invasive lobular carcinoma (ILC), representing 5–15% of all breast cancers^1^,^2^. ILC can be subdivided into five more specific histological subtypes^3^. ILCs are typically oestrogen receptor (ER) and/or progesterone (PR) positive and exhibit frequent loss of expression of the cellular adhesion molecule E-cadherin (CDH1)^1^. A subset of ILCs is HER2 positive. ILCs have very similar survival to IDCs at both five and 10 years, but despite this similar survival, the clinical course is distinct: ILCs are three times more likely to metastasize to the peritoneum, gastrointestinal tract, and ovaries and are more frequently bilateral^4^, pointing towards differences in underlying biology.

Gene expression-based molecular subtypes have been used as a reference to describe breast cancers^5^,^6^. Such subtypes are relatively well reflected in the immunohistochemistry (IHC)-based diagnosis used in the clinic^7^. However, they were mainly defined based on IDCs. Some molecular studies have been performed on ILC, using comparative genomic hybridization^8^ or gene expression profiling^9^, and more recently targeted DNA sequencing in advanced disease^10^. Two recent studies extensively characterizing large breast cancer cohorts^11^,^12^ contain ILCs, but are dominated by IDCs, leaving ILC largely uncharacterized^13^. The Cancer Genome Atlas (TCGA) consortium recently analysed 127 ILC tumours in comparison to 490 IDC tumours^14^. Focusing on 106 luminal A ILC samples, they defined three subtypes termed Reactive-like, Immune-related and Proliferative. Most of their molecular analyses focused on contrasting ILC to IDC tumours.

Treatment decisions made by oncologists for breast cancer are mainly based on results obtained in large trials, in which ILCs are only a minor subgroup. It is, therefore, not always the case that the conclusions from “breast cancer” trials also apply to ILC.

As part of the Rational Therapy for Breast Cancer (RATHER) consortium (www.ratherproject.com), we set out to improve the molecular characterization of ILCs by searching for potential molecular subtypes and oncogenic driver events. In addition, we aimed to understand the molecular events leading to different clinical outcomes. We collected a large cohort of 144 ILC patients with complete clinical data and long follow-up, and performed comprehensive molecular profiling of their primary tumour. The integration of multiple molecular data reveals two distinct molecular subtypes of ILC and provides new insights into the molecular factors associated with this disease.

## Results

### Molecular profiling of ILCs

To explore the biology of invasive lobular carcinomas (ILCs), we performed comprehensive molecular profiling of 144 untreated tissue samples from primary ILC tumours with 6.8 years median clinical follow-up (Additional file 1) using (i) targeted DNA sequencing to study somatic variants on a set of 613 genes (518 protein kinases and 95 additional cancer genes, Additional file 2); (ii) SNP6 arrays to study somatic copy number alteration (CNA) profiles; (iii) DNA microarrays to study gene expression and (iv) reverse-phase protein arrays (RPPA) to measure the expression of 168 selected proteins and phospho-proteins (Additional file 3). For 131 samples (91% of samples profiled), we obtained DNA sequencing, CNA and gene expression data (Figure S1A), 112 of which also have RPPA data (85%). Most of the samples are ER/PR positive based on immunohistochemistry and only one sample does not show evidence of hormone receptor expression (Figure S2).

### Identification of two subtypes of ILC

**Figure 1:**
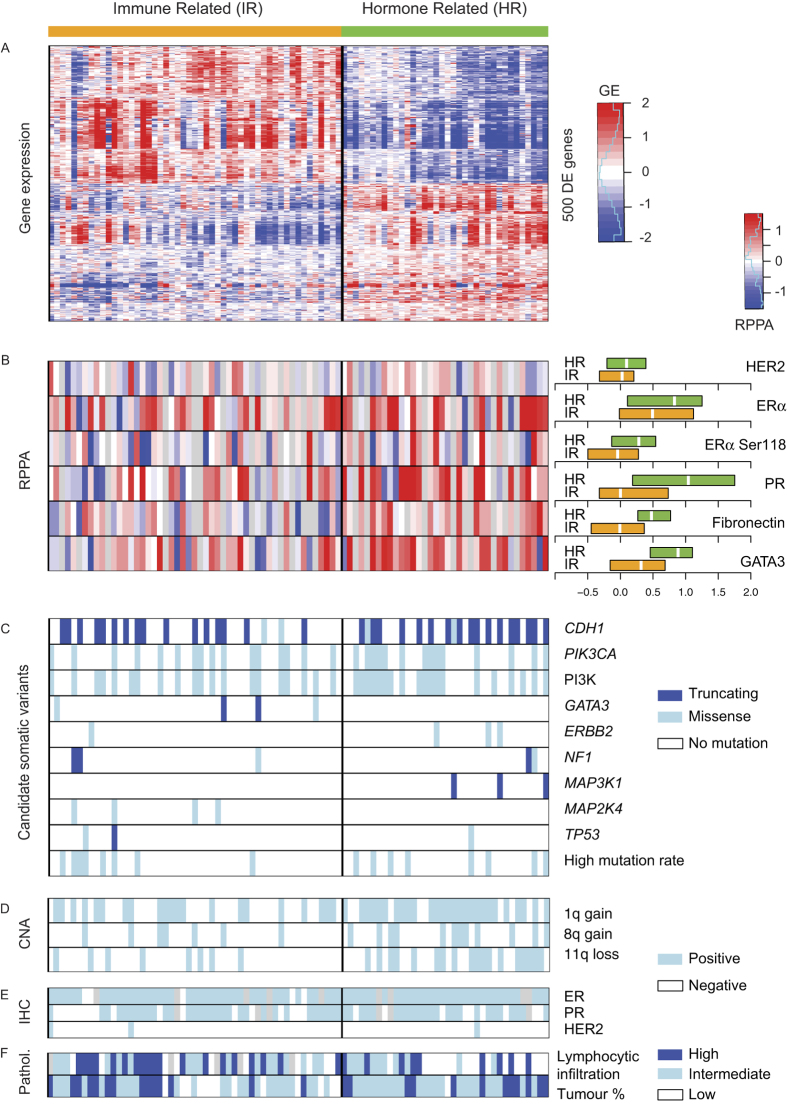
Gene expression clustering reveals two ILC subtypes. We defined two robust clusters of ILC samples by consensus clustering on the genome-wide gene expression data: immune related (IR) and hormone related (HR). We represent here the 89 samples with DNA sequencing, CNAs, and gene expression (Figure S1B). (**A**) Gene expression of top 250 up-regulated and top 250 down-regulated genes in one subtype versus the other. (**B**) RPPA values of selected epitopes. The boxplots on the right represent the distributions in both subtypes. (**C**) Candidate somatic variants are indicated in blue (truncating mutations in dark blue and missense mutations in light blue), while white indicates the absence of variant. PI3K is blue when any of the PI3K pathway genes is mutated (Figure S12). Samples with a high somatic mutation rate (> = 10) are shown in blue (white otherwise). (**D**) Copy number of selected genes. Presence (resp. absence) of the given CNA is shown in light blue (resp. white). (**E**) ER, PR and HER2 status as assessed by immunohistochemistry (IHC). (**F**) Pathology assessment of lymphocytic infiltration (defined with 3 levels) and tumour cellularity (High is >70%; Intermediate is (40–70%]; low is [30–40%]). Light blue (resp. white) indicates positive (resp. negative) and grey represent missing values in (**B,E**,**F**).

Extensive stability analysis of clustering of the gene expression data using a variety of clustering approaches identified two robust expression subtypes (Fig. 1A, Figure S3). Based on a gene sub-sampling analysis, we could stably classify 71% (102/144) of the samples into one of two subtypes comprising 63 and 39 samples, respectively. Among these 102 samples, 89 have mutation, CNA and gene expression data (Figure S1B) and are represented in Fig. 1.

**Figure 2:**
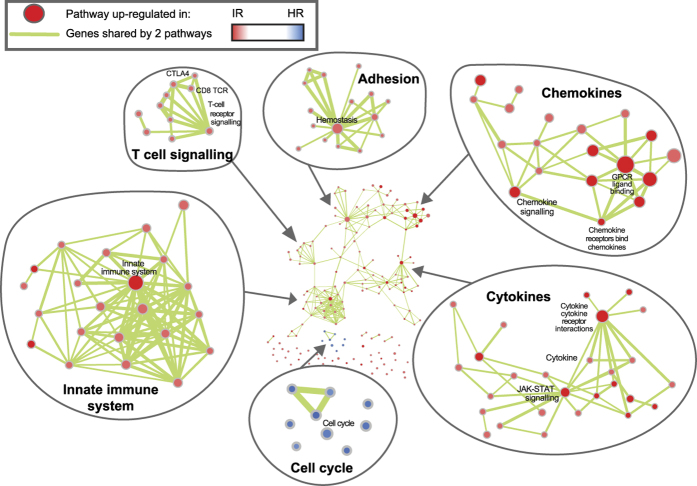
Pathway Enrichment Map contrasting both subtypes. The networks illustrate the results of the pathway enrichment analysis (GSEA) contrasting IR and HR subtypes. Each node represents a pathway. Links between nodes represent the genes shared by both pathways (overlap coefficient >0.5). The node colours represent the strength and direction of the enrichment (red pathways are up-regulated in IR, blue ones are up-regulated in HR). The figure was made with the Enrichment Map app^15^ from Cytoscape^45^.

**Figure 3:**
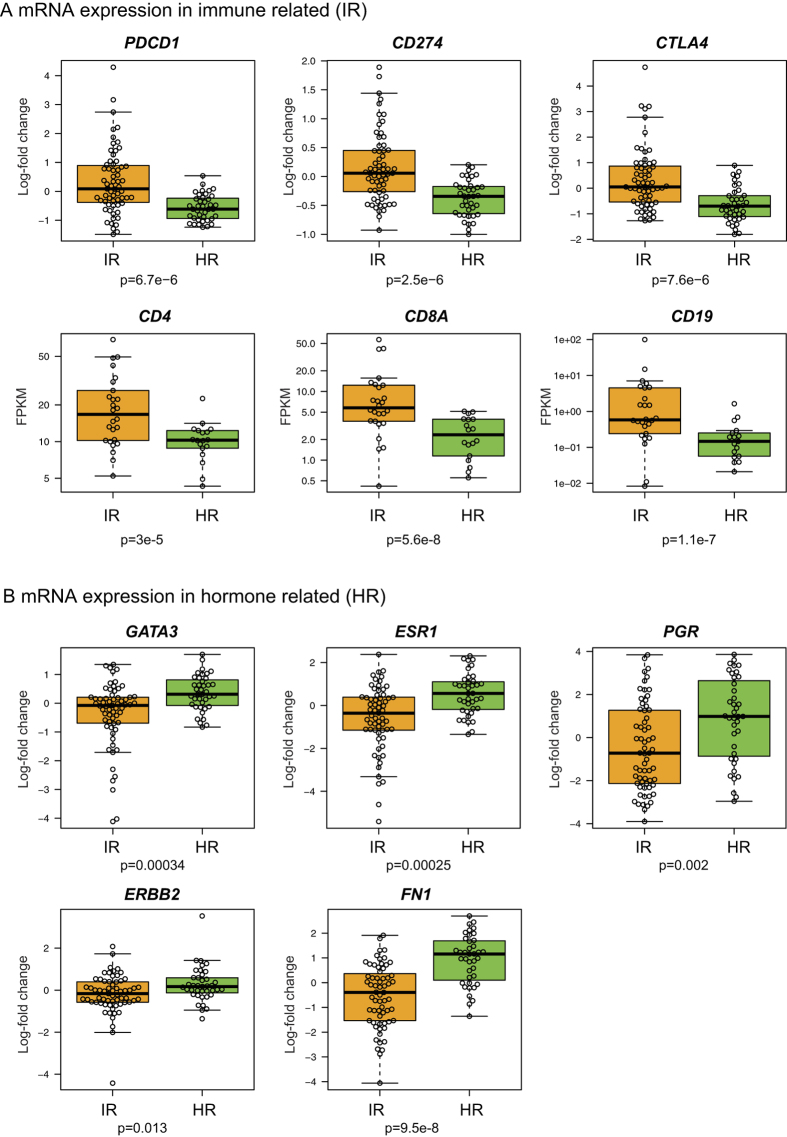
Gene expression of subtype biomarkers. The boxplots show the normalized gene expression in both IR and HR subtypes for different genes from the microarray data, unless otherwise specified. *CD4, CD8*A and *CD19* absolute levels were quantified with RNA sequencing data on a subset of 68 samples and shown here by the number of Fragments Per Kilobase per Million (FPKM). (**A**) Biomarkers of the IR subtype: negative regulators of the immune response, T-cell markers *CD4* and *CD8A*, and B-cells marker *CD19* are up-regulated in IR. *CD19* is only lowly expressed (FPKM < 1 in most samples). (**B**) Biomarkers of the HR subtype. Differences are assessed by a Wilcoxon’s rank sum test, except for the RNA sequencing data where the p-value is derived from differential expression analysis using DESeq2^45^.

Pathway analysis revealed specific biological processes associated with each subtype (Additional file 4). The first, referred to as Immune Related (IR), shows up-regulation of genes characteristic of cytokine/chemokine signalling. Representing the enriched pathways as nodes in an Enrichment Map^15^, we identified a striking enrichment for chemokines, cytokines and innate immune signalling (Fig. 2). The IR subtype shows up-regulation of a range of lymphoid signalling molecules at the mRNA level (e.g. TGFBR2, IL11RA, TNFRSF17, CCL15, CCL14, CCR2, CD27, XCL2, IFNAR2, CD40LG are the 10 most up-regulated genes in the cytokine-cytokine receptor interaction pathway), suggesting alterations in either the composition or functional activity of immune cells within these tumours. Interestingly, negative regulators of the immune response *PDCD1* (PD-1), *CD274* (PD-L1) and *CTLA4* (CTLA-4) are expressed at higher mRNA levels in the IR subtype (Fig. 3A). The IR subtype is associated with METABRIC IntClust 4^11^, characterized by lymphocytic infiltration (Table S1). In addition, pathology assessment of all the slides showed that the IR subtype shows intermediate to severe lymphocytic infiltration in 78% of the samples, as compared to 55% for the other subtype (p = 0.022, Fisher’s exact test, Table S2, Fig. 1F). Moreover, T-cell markers *CD4* and *CD8A* are significantly up-regulated at the mRNA level in the IR subtype, while the B-cell marker *CD19* does not seem to be expressed (Fig. 3A), suggesting that the immune infiltrate in the IR subtype is enriched in T-cells, which was further supported by CD4 and CD8 staining data (Figure S4).

The second subtype, referred to as hormone related (HR) subtype, shows higher levels of oestrogen (*ESR1*) and progesterone (*PGR*) receptors (Fig. 3B) and up-regulation of cell cycle genes (Fig. 2) and oestrogen receptor (ER) target genes (Additional file 4, Figure S9). We also observed higher expression of luminal B signature genes, consistent with up-regulation of cell cycle genes. Using a set of ER-responsive genes identified in MCF-7 cells^16^, we found that 685 of the 1902 signature genes were differentially expressed, many more than expected by chance (binomial test, p < 1e-6). This finding was supported by mRNA (Fig. 3B) and protein epitope analysis (Fig. 1B), which confirmed higher expression of ER, PR, and phosphorylated ER (Ser118). Even though almost all tumours are ER and PR positive by IHC (Fig. 1E), we find a higher level of hormone receptor expression in the HR subtype (Fig. 3B). In addition, GATA3, an important player in ER signalling^17^,^18^, shows up-regulation at both gene expression and protein levels in the HR subtype (Figs 1B and 3B). Collectively, these findings support elevated levels of hormone receptor signalling in the HR subtype.

### The two ILC subtypes are validated in two independent datasets

To validate the existence of IR and HR ILC subtypes, we investigated the ILC samples of the METABRIC consortium^11^ and the TCGA breast cancer. Using the same clustering approach *de novo* on the validation gene expression data, we also identified two robust subtypes (Figure S5). We found that differential gene expression between these subtypes was correlated with differential gene expression between the IR and HR subtypes in the RATHER dataset (Figure S6). Moreover, both METABRIC and TCGA subtypes displayed similar differences in biological processes (Figure S7). In particular, processes up-regulated in METABRIC subtype 1, which is most similar to the IR subtype, include cytokine receptor interaction, collagen-related processes, adhesion and extra-cellular matrix (Additional file 4). We also found negative regulators of immune response up-regulated in this subtype (Figure S8). Cell cycle, *ESR1* targets and luminal B signature genes were up-regulated in METABRIC subtype 2, consistent with our findings in the HR subtype (Figure S9).

We investigated the overlap between the IR/HR subtypes and the subtypes defined by TCGA (Reactive-like, Immune-related and Proliferative) on the TCGA and METABRIC samples. On the TCGA samples, the Reactive-like subtype is associated with the IR subtype, while Immune-related and Proliferative subtypes are associated with the HR subtype (p-value < 1e-6). On the METABRIC samples, the subtypes do not show this association (p-value = 0.47) (Table S3A). The differences could come from the fact that the TCGA subtypes were derived on luminal A ILCs specifically. However, luminal A samples were equally distributed between IR and HR subtypes and clustering only the luminal A samples, we recovered the IR and HR subtypes (Table S4). Thus, IR and HR are distinct subtypes that do not reflect previous classifications.

### Genomic markers of each subtype

Genomic profiling was performed to identify mutations in kinases and breast cancer genes that contribute to the development of ILC. A small capture library was used to ensure high-level coverage to account for low tumour cellularity. DNA sequencing identified 887 candidate somatic variants that were predicted to alter protein sequence and were not present in catalogues of germline variants or a panel of normal samples. The median number of variants per sample was six, corresponding to 1.8 variants per Mb. A subset of samples displayed a higher mutation rate. Frequently mutated genes include *CDH1* (42.8%), *PIK3CA* (34.8%), *GATA3* (5.1%), *MAP3K1* (5.1%), and *AKT1* (5.1%) (Figure S10, Additional file 5). As expected, *CDH1* mutants show lower expression at both the mRNA (Wilcoxon p = 2.4e-5) and protein (Wilcoxon p = 8.9e-4) levels (Figure S11). However, we also observe *CDH1* WT samples with low mRNA and protein levels, pointing towards other inactivating mechanisms, as previously described^19^. Genes with low mutation frequencies (<5%) include *MAP2K4* (4.3%), *NF1* (4.3%), *ERBB2* (4.3%) and *TP53* (3.6%) (Fig. 1C). The PI3K pathway was mutated in 46% of the tumours, with mutations in *AKT1* (5.1%), *PIK3R3* (2.9%), *PTEN* (1.4%), *PIK3CB* (1.4%), *PIK3CG* (1.4%), *PIK3CD* (1.4%) and *PIK3CA* that tended to be mutually exclusive (Figure S12). This confirms a central role for the PI3K pathway mutations in the development of ILC^20^, and was found in both subtypes. Most *ERBB2* mutations occur in the HR subtype (Fig. 1C). *GATA3* mutations are enriched in the IR subtype, even though not significantly so (Fig. 1C, Table S5) and are mostly inactivating. GATA3 is critically required for the activation of the ER pathway in response to oestrogen^17^,^18^, which is consistent with impaired ER/PR signalling in the IR subtype.

We performed high-density SNP genotyping and identified recurrent copy number alterations (CNAs) using ADMIRE^21^. We found 158 regions recurrently altered in the 22 pairs of autosomal chromosomes (Additional file 6), including gains of chromosomes 1q, 5q, 8q, 16p and 20q and loss of chromosomes 1p, 6q, 11q, 13q, 16q and 18q (Figure S13) consistent with previous studies^1^. Comparing the occurrence of recurrent CNAs within both subtypes, we found gain of chromosomes 1q and 8q and loss of chromosome 11q to be more frequent in the HR subtype (Fig. 1D, Table S5).

### OncoScape data integration identifies candidate drivers

To identify potential drivers of both subtypes, we used OncoScape, a tool for prioritizing oncogenes and tumour suppressors by integrating multiple data types (submitted). Comparing mutations, CNAs, gene expression and RPPA between both subtypes, we found four potential drivers of the HR subtype (Additional file 7): *PGR, GATA3* and *FN1*, which are up-regulated at the mRNA and RPPA level, corroborating our previous results (Figure S14), but also *YAP1*, which is deleted and down-regulated in HR. Lehn *et al.* showed that *YAP1* down-regulation leads to over-expression of ER and PR *in vitro* and was associated with tamoxifen resistance in a primary breast cancer cohort^22^. This suggests that *YAP1* down-regulation could contribute to elevated hormone signalling in the HR subtype. We did not identify specific drivers for IR, which may be due to the lack of immune signalling representation in the RPPA data.

### Modelling therapeutic response in cell line models

Both IR and HR subtypes show similar clinical outcomes (Figure S15). To identify candidate therapeutic options, we profiled a set of 15 ILC-like cell lines. Since there are relatively few good ILC breast cancer cell lines, we gathered the best available cell lines. More specifically we selected cell lines with inactivating mutations in CDH1 (E-cadherin) and CTNNA1 (α-catenin) resulting in inactivation of the complex these proteins belong to. We also employed gene expression profiles to verify that the cell lines resemble the subtypes and used these profiles to map the cell lines to the IR and HR subtypes (Figure S16). We then used the response data for 88 drugs^23^ on a subset of these cell lines to test for differential drug sensitivity between the subtypes (Additional file 8). We retained six drugs showing differential response at an FDR < 0.25 (Figure S17). Cell lines of the IR subtype are more sensitive to three different DNA-damaging agents: Bleomycin, Cisplatin and the topoisomerase 1 inhibitor SN-38.

### EMT segregates the ILC samples

**Figure 4:**
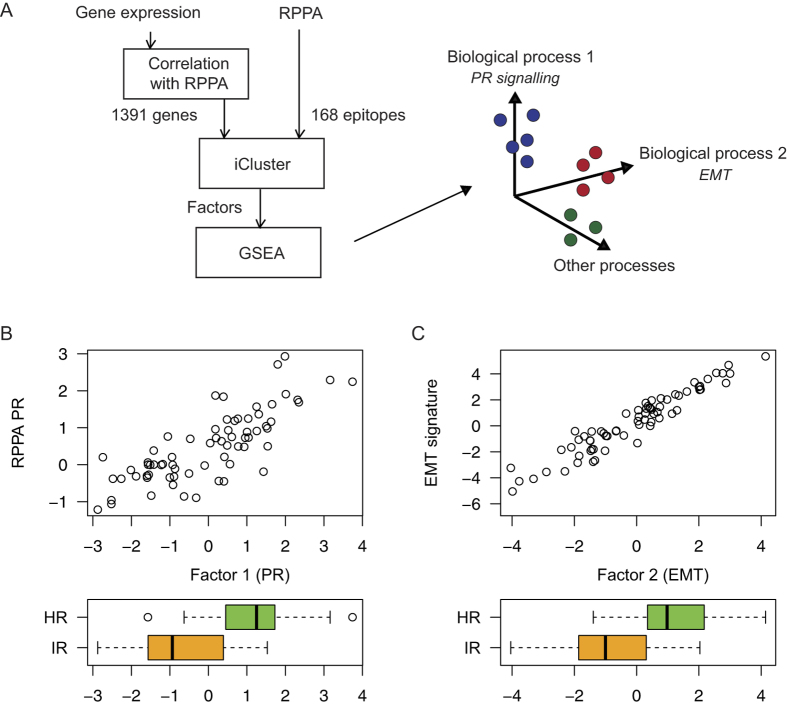
Factor analysis of mRNA and protein expression. (**A**) Integrative analysis of gene expression and RPPA data using iCluster to identify factors best characterizing the samples. (**B**) The second factor is highly correlated with PR (from RPPA) and higher in the ER/PR subtype. (**C**) The first factor is highly correlated with the EMT gene expression signature of Anastassiou *et al.*^26^, and higher in the ER/PR subtype.

Performing an integrative analysis of the RPPA and gene expression data using iCluster^24^, we identified five main factors characterizing the samples (Fig. 4A). Next, we performed a gene set enrichment analysis^25^ to determine the biological processes associated with each factor. The first factor is associated with progesterone receptor (PR) signalling and correlates well with the PR levels in the RPPA data (Fig. 4B). This supports the validity of the HR subtype we identified. Not surprisingly, we find that samples in the HR subtype have higher values of this factor. The second factor is associated with the epithelial-mesenchymal transition (EMT) and highly correlates with the top two genes (*COL11A* and *THBS2*) in the EMT signature defined by Anastassiou *et al.*^26^ (Fig. 4C). Interestingly, we found that the HR subtype has significantly higher EMT scores (p < 1e-6, Fig. 4C) and fibronectin is up-regulated at both mRNA and protein levels in these samples (Figs 1B and 3B).

### Molecular markers associated with survival in ILC

To assess patient outcome, we performed Cox proportional hazards regression, stratified by biobank and fitted with commonly used clinical variables (Table S6). Tumours with a high number of non-silent somatic mutations were associated with poor survival (Figure S18), with an adjusted Hazard Ratio of 1.26 (95% CI 1.10 to 1.45). Notably, at 10 years after diagnosis, approximately 80% of the patients with low mutation rates are alive, compared to only 45% of the patients with high mutation rates. In addition, 18 proteins and phospho-proteins were found to be associated with survival (Additional file 9). Two had an FDR < 1%: a higher level of eIF4B was associated with poor survival, (Figure S19A, adjusted Hazard Ratio 10.35, 95% CI 2.5 to 42.88) while a higher level of histone H2AX was associated with better survival (Figure S19B, adjusted Hazard Ratio 0.3376, 95% CI 0.16 to 0.73).

### High mutation rate and eIF4B level stratify patients into three distinct outcome groups

**Figure 5:**
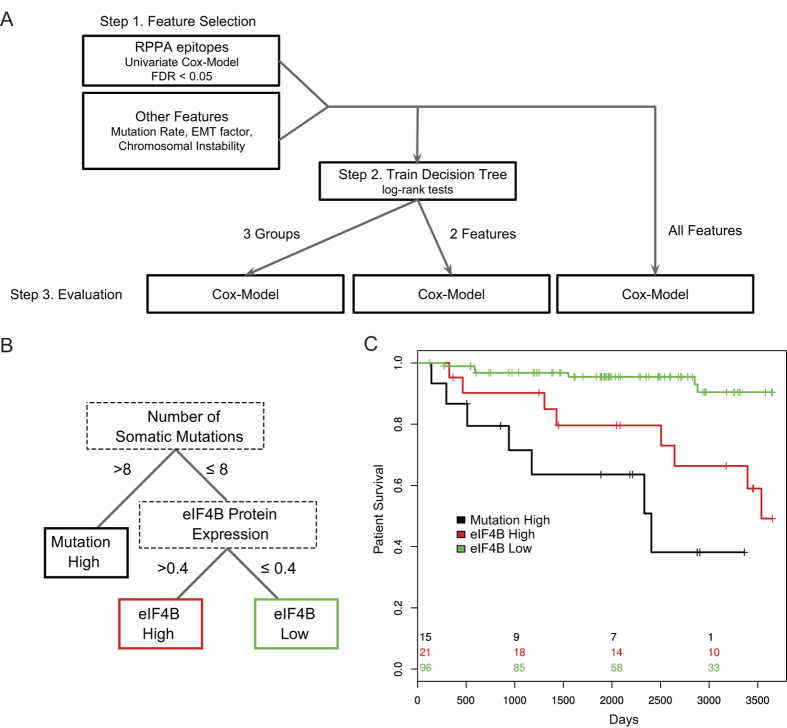
Survival tree. (**A**) Workflow of the approach to predict survival from multiple data types. (**B**) The resulting decision tree, classifying the samples based on their somatic mutation rate and eIF4B protein level. (**C**) Kaplan-Meier curves of the groups of samples defined by the decision tree. Samples with high mutation rate have a poor survival, while samples with low eIF4B level have a good survival.

To combine the various predictive signals, we trained decision trees based on RPPA epitopes, the EMT factor, mutation rates and chromosomal instability (Fig. 5A). We found a decision tree based on mutation rate and eIF4B protein expression to be highly predictive of survival in the presence of clinical variables (Fig. 5B): patients with high mutation rate show poor survival while patients with low eIF4B level exhibit good survival (Fig. 5C). During the internal cross-validation of the different models, the same features (mutation rate and eIF4B level) were selected by the decision tree in most folds and performed well (Figure S20), highlighting their robustness. After adding commonly used clinical variables to the inputs, we found a similar tree with lymph node count as an additional predictive feature, which identified a few patients with a very high number of positive lymph nodes and poor survival. Removing these patients further improves the predictive quality of the good prognosis factors with only two events in this group (Figure S21).

eIF4B is required for the binding of mRNA to ribosomes and functions in association with the translation initiation complex^27^ and was shown to predict poor outcome in ER negative breast cancer^28^. Moreover, Choi *et al.* recently showed that eIF4B phosphorylation is responsible for the increase in CIP2A translation, a key factor in estradiol-enhanced proliferation^29^, indicating a possible link with hormone-dependent tumour growth. When we investigated the influence of therapy, the patients with low mutation rate and high eIF4B appeared on the Kaplan-Meier plot to be highly responsive to anti-hormonal therapy (Figure S22). Even though the small size of the subsets precludes significance to be achieved in statistical tests, this result suggests that high eIF4B could increase proliferation, leading to poor survival, but this can be effectively controlled by anti-hormonal therapy.

## Discussion

**Figure 6:**
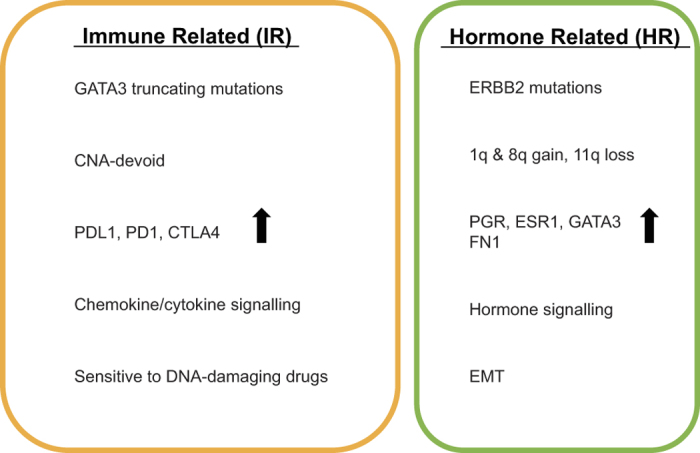
Summary description of both subtypes. The figure represents the immune related (IR) and hormone related (HR) subtypes with their main characteristics.

In this study we provide a detailed analysis of biological processes in ILC. We integrated genomic, proteomic, transcriptomic and clinical data for this specific subgroup of breast cancers. Unsupervised clustering of the genome-wide gene expression revealed two subtypes: an immune related subtype, characterized by lymphocytic infiltration and up-regulation of “checkpoint proteins”, and a hormone related subtype, characterized by active ER/PR signalling and EMT (Fig. 6). Similar to the recent TCGA study^14^, we found that ILCs frequently carry mutations that inactivate *CDH1* and that activate the PI3K pathway. Thus, PI3K pathway inhibitors may represent a plausible treatment option for ILC patients. We also found low frequency mutations in a number of signalling molecules including *ERBB2, MAP3K1*, and *MAP2K4* and low frequency inactivating mutations in *TP53* and the transcription factor *GATA3*, which is critically important for ER signalling.

ILCs often show a characteristically diffuse pattern of growth and clinical samples often have a relatively low tumour content. To rule out the possibility that the two identified subgroups were consequence of different tumour cellularity, we repeated the clustering using samples with high tumour cellularity (>50%). This analysis robustly identified the two subtypes (Table S7). In addition, we find that the noise level is similar in the subtypes and that we detect sizeable CNAs in both HR and IR subtypes (Figure S13). To investigate whether the tumours with high immune gene expression (as represented by CD8A expression) show low levels of GATA3, ESR1 and PGR, we scattered each of these proteins against CD8A RNAseq counts (Figure S23). We observe both high and low protein expression at both high and low CD8A mRNA expression levels. Thus, while CD8A, ESR1, PGR and GATA3 are all individually associated with the subtypes, this association does not arise due to the different levels of immune cells in the subtypes.

The IR subtype showed high expression of numerous cytokine/chemokine signalling pathway components found in lymphoid cells, and over-expression of *CD4* and *CD8A*. In both ER-negative and ER positive/HER2 positive breast cancer, the presence of CD8+ T cells was shown to be associated with a significant reduction in the relative risk of death from disease^30^. However, the IR tumours also had high expression of the negative regulators of immune response *PDCD1* (PD-1), *CD274* (PD-L1) and *CTLA4* (CTLA-4). We observed some diffuse staining in both tumour and immune cells in some of the samples (Figure S24). These IR tumours likely have an immune infiltrate and blocking these active checkpoints might reactivate the immune response. Antibodies targeting PD-1, PD-L1 and CTLA4 have recently shown promise in the context of metastatic melanoma and cancers with mismatch repair deficiency and are now entering trials for breast cancer. We note that some tumours in the HR subtype show high lymphocytic infiltration and may also benefit from immune-based therapy. Interestingly, we find that cell lines representing the IR subtype show better response to DNA-damaging agents. As the limitations of cell lines as models for tumour drug response are well known, this finding requires further validation. Interaction between therapy and the immune system has been shown to result in better clinical outcome^31^,^32^. Lymphocytic infiltration and tumour-infiltrating-lymphocytes were associated with better response to (neo)adjuvant chemotherapy^33^,^34^. This suggests that the immune component of the IR subtype could positively interact with chemotherapy and benefit the patients. Since it remains unclear which ILC patients will benefit the most from chemotherapy, this may have implications for the selection and dosing of therapy for these patients. It is tempting to speculate that the IR subtype may further benefit from combined treatment with DNA damaging agents and immunomodulatory agents.

In addition to the two ILC subtypes that we characterized, we also found associations between molecular features of ILC and clinical outcome: based on the mutation rate and eIF4B level of the tumours, we were able to distinguish three subgroups with different prognosis. Together, these characterizations may help to guide the treatment of ILC through the identification of patients that may benefit from specific targeted, chemo- and/or immune-therapies. Broader genomic profiling will be important to understand the molecular factors contributing to the high mutation rate observed in some patients.

## Methods

Some more details are given in the Supplementary Information file and data access is provided in the Data availability section.

### Clinical data

All patients with an ILC treated in the NKI-AVL since 1980 and in the Addenbrookes Hospital Cambridge UK since 1997 with available fresh frozen (FF) material were extracted from the hospital database. We also sourced FF tissue from adjacent matched normal tissues when available (n = 55). Subsequently, we collected matched formalin fixed paraffin embedded (FFPE) tissue blocks for TMA construction. The NKI-AVL and Cambridge medical ethical committees approved the study and the use of anonymized archival tissue in this study. All experiments were performed in accordance with relevant guidelines and regulations. For the Cambridge samples, the study was approved by ‘NRES, Cambridgeshire 2 Research Ethics Committee’ (project number 07/H0308/161). Following the Dutch national guidelines on the use of left-over tissue and exceptions/clarifications in Dutch national law, the NKI-AVL ethical committee allowed performing the study without collecting informed consent, provided that patient data would be anonymized (decision letter July 29th 2010). This decision was reviewed by ethical expert Prof. Elaine Kay. Following the WHO definition of ILC, we used a morphological diagnosis to select the samples. Tumour samples were centrally revised for tumour percentage of the FF material, histological grade on FFPE and ILC sub-classification^3^ on FFPE. TMAs were stained for ER, PR and HER2. Samples were defined to be ER-positive or PR-positive when 10% or more of the tumour cells showed positive staining of ER or PR respectively based on immunohistochemistry. We note that this was the standard European threshold at the time the samples were collected. Using a cut-off of 1% would change the ER and PR status of one and 12 tumours, respectively (quantitative values are available in the data availability section). One sample (not in the subtypes) was HER2 positive (intensity of 3) and four samples (2 in IR, 2 in HR) were uncertain (intensity ~2). For survival analysis, we considered only breast cancer specific survival, due to the presence of competing events and (distant) recurrence free survival. For patient stratification in Kaplan-Meier plots, we used Kaplan-Meier estimator and calculated p-values with the log-rank test. Cox proportional hazards regression model was stratified by biobank and, unless otherwise specified, fitted with commonly used clinical variables: tumour size, grade, number of positive lymph nodes, treatment and age at diagnosis. Association of a variable with survival was tested with a likelihood-ratio test comparing a model including clinical variables over a model including clinical variables and the variable tested.

### Affymetrix SNP 6.0 arrays

As presented earlier^11^, each sample was preprocessed using the PennCNV pipeline for Affymetrix arrays^35^. Genotyping calls were obtained with Affymetrix Power Tools software using the Birdseed algorithm. Each array was wave-corrected using the built-in algorithm in ASCAT v.2.2^36^ and copy numbers were called with ASCAT v2.2 using information from the matched normal when available. The samples were classified into the 10 integrative clusters from METABRIC using the iC10 package^37^. We applied ADMIRE^21^ to identify recurrent alterations.

### DNA Sequencing

DNA sequencing was performed on an Illumina HiSeq 2000 platform. For each sample, Illumina TruSeq index libraries were constructed according to manufacturer’s instructions before being enriched by capture with a biotinylated RNA probe set targeting the human kinome and a range of cancer related genes (Agilent Technologies, 3.2 Mb). We sequenced 10 to 12 samples on a single Illumina HiSeq 2000 lane to generate 50 bp paired-end reads. On average, we obtained 26,985,771 unique reads on each run. The average kinome coverage (mean bait coverage) for the whole sequencing dataset is 133X, ranging from 36 to 258. On average, 91% of the target positions are covered by 20x. We aligned the raw sequencing data with the Burrows-Wheeler Aligner (BWA) version 5.10, backtrack algorithm, to the human genome (Ensembl 37) removing duplicate reads and reads with mapping quality <60. Single nucleotide variants and indels were called using SAMtools on unique paired aligned data. We used dbSNP and variant data from the Exome Variant Server together with a pool of normal 80 DNAs taken from various tissues to remove potential germline variants. A set of candidate somatic variants was selected for validation by sequencing matched tumour and normal material. Variants found back in the tumour sample and not in the normal are validated mutations (VALIDATED); variants found in both the tumour and the normal samples are rare germline variants (SNP); variants not found back in the tumour samples are false positive calls (ABSENT); finally some variants were tested but the experiment failed (FAILED).

### Microarray hybridization

The RNA for microarray analysis and sequencing was purified using the Qiagen RNeasy micro kit according to manufacturer’s protocols. RNA was amplified, labelled and hybridized to the Agendia custom-designed whole genome microarrays (Agilent Technologies) and raw fluorescence intensities were quantified using Feature Extraction software according to the manufacturer’s protocols. We performed background subtraction using an offset of 10. All probe intensities <1 were set as missing values. The log2 transformed probe intensities were quantile normalized^38^ using limma. Batch-effects were adjusted for using ComBat^39^. Genes with multiple probes were summarized by the first principal component of a correlating subset.

### Gene expression clustering

We applied several different clustering algorithms on the top 1000 genes with highest median absolute deviance: hierarchical clustering with Pearson distance and ward D1, single, average and complete linkage, as well as non-negative matrix factorization (NMF). The ward D1, average and NMF methods returned stable clustering results as assessed by consensus clustering. All three methods found largely the same two clusters (Figure S3). To define subtypes, we first performed consensus clustering with average linkage, two clusters, and 90% feature resampling. Then, the consensus matrix was hierarchically clustered with complete linkage and Euclidean distance. We defined two clusters, such that all samples within each cluster had a pairwise concordance of at least 80%. Samples not falling into one of these two clusters were not assigned to any cluster (n = 42).

### Validation datasets

We used the ILC samples of METABRIC^11^ as a validation set for the gene expression subtypes. 76 samples are in common between RATHER and METABRIC (Additional file 10) and were removed from the validation set, resulting in a set of 103 samples. We mapped probes to genes with the ReMOAT annotation^40^. We downloaded TCGA RNAseq data for 187 ILC samples on Feb 4^th^, 2014.

### Reverse Phase Protein Arrays

Three sections of fresh frozen tissue were lysed in hot Laemmli buffer (50 mM Tris pH = 6.8, 2% SDS, 5% glycerol, 2 mM DTT, 2.5 mM EDTA, 2.5 mM EGTA, 1× HALT Phosphatase inhibitor (Perbio 78420), Protease inhibitor cocktail complete MINI EDTA-free (Roche 1836170, 1 tablet/10 mL), 2 mM Na_3_VO_4_ and 10 mM NaF) and boiled for 10 min at 100 °C. Samples were sonicated for 1–2 min to break the DNA and spun for 10 min at 13,000 rpm. Supernatant was snapfrozen and protein concentration was measured (BCA reducing agents compatible kit, Pierce, Ref 23252). Samples with sufficient protein concentration (>0.5 mg/ml) were printed onto nitrocellulose-covered slides (Sartorius, Grace Biolabs or Maine Manufacturing) using a dedicated arrayer (2470 Arrayer, Aushon Biosystems) in five serial dilutions (0.5 to 0.03125 mg/ml) and two technical replicates. Arrays were labelled as described in Rondeau *et al.*^41^. Specificity of each primary antibody was first validated by Western blotting on a panel of breast cancer cell lines. Data were normalized using Normacurve software^42^. Bias due to origin of the samples (NKI vs CAM) was removed using a median regression approach.

### Drug sensitivity

We profiled a panel of 15 ILC-like cell lines (Figure S16). Drug sensitivity was assessed on the Sanger cell line panel. Among the 262 drugs, we focused our assessment on 88 agents that had measurement in at least three cell lines per subtype. With this dataset, we performed a two-sided t-test between the AUC of the dose-response curves of the cell lines in the two subtypes, correcting for multiple testing with the Benjamini-Hochberg method. We show the IC50 on Figure S17 for easier interpretation.

### Gene expression and RPPA integration

We applied a factorization integrating RPPA and gene expression data, and then did a gene set enrichment analysis (GSEA)^25^ on these factors. To extract concordant data for the factorization, we selected only the expression of the 1391 genes that were in the top 10 correlating (absolute Pearson’s ρ) with any RPPA epitope. All RPPA epitopes were used. The iCluster method^24^ was re-purposed to perform factorization. The weights of the features for each factor are provided in Additional file 11. For pathway analyses we used the mSigDB v4.0 ‘canonical pathways’ (called pathways) and ‘chemical and genetic perturbations’ (called signatures) gene set collections. The gene expression signature defined by Anastassiou *et al.*^26^ was significantly associated with one of the factors, thus interpreted as representing EMT.

### Gene expression subtype pathway analysis

To contrast the IR and HR subtypes we also used GSEA. Genes were ranked by differential expression (signal-to-noise ratio) between the two clusters. To specifically investigate oestrogen signalling, we used the list of up and down regulated genes upon oestrogen stimulation of MCF-7 cells as identified by Zwart *et al.*^16^.

### Decision tree

Decision trees were built using conditional inference trees^43^. We applied Bonferroni correction and used a p-value threshold of 0.25, a minimum of 20 samples to split, and a minimum of 10 samples in a leaf node. As input features for the tree, we considered i) mutation and CNA rates as a summary for the level of genetic instability; ii) the EMT factor, which was the strongest component of the integrated analysis of gene expression and RPPA data and iii) the epitopes from RPPA that showed a significant association with survival with a likelihood-ratio test. The thresholds we used to define the final tree are based on a tree trained with clinical variables as additional variables. Performance of the tree was assessed by partial likelihood deviance based on leave-one-out cross-validation (Figure S20).

### Data availability

Public data access to the raw data is available with the following DOIs:

- Clinical data:
  1. http://dx.doi.org/10.6084/m9.figshare.1301848
- Immunohistochemistry data:
  1. http://dx.doi.org/10.6084/m9.figshare.1360201
- Variants data:
  1. http://dx.doi.org/10.6084/m9.figshare.1373846
- Copy number data:
  1. http://dx.doi.org/10.6084/m9.figshare.1314577
- Gene expression data:
  1. Gene Expression Omnibus (GEO) accession GSE68057
  2. http://www.ncbi.nlm.nih.gov/geo/query/acc.cgi?acc=GSE68057
- RPPA data:
  1. Gene Expression Omnibus (GEO) accession GSE66647
  2. http://www.ncbi.nlm.nih.gov/geo/query/acc.cgi?acc=GSE66647

## Additional Information

**How to cite this article**: Michaut, M. *et al.* Integration of genomic, transcriptomic and proteomic data identifies two biologically distinct subtypes of invasive lobular breast cancer. *Sci. Rep.* **6**, 18517; doi: 10.1038/srep18517 (2016).

## Supplementary Material

Supplementary Information

Patient Table

Kinome target genes

RPPA epitopes

GSEA results

Mutated genes

Recurrent CNA

OncoScape

Differential drug response

RPPA survival

METABRIC and RATHER-samples

Factors components
