# Supplementary material for: Integration of genomic, transcriptomic and proteomic data identifies two biologically distinct subtypes of invasive lobular breast cancer
Source: Sci Rep. 2016 Jan 5;6:18517. doi: 10.1038/srep18517 (PMC4700448; doi:10.1038/srep18517)
Supplement: Supplementary Information [file srep18517-s1.pdf]

Integration of genomic, transcriptomic and proteomic data identifies two biologically distinct subtypes of invasive lobular breast cancer

Supplementary Information

Magali Michaut[*], Suet-Feung Chin[*], Ian Majewski[*], Tesa M. Severson[*], Tycho Bismeijer[*], Leanne de Koning[*], Justine K. Peeters[*], Philip C. Schouten, Oscar M. Rueda, Astrid J. Bosma, Finbarr Tarrant, Yue Fan, Beilei He, Zheng Xue, Lorenza Mittempergher, Roelof J.C. Kluin, Jeroen Heijmans, Mireille Snel, Bernard Pereira, Andreas Schlicker, Elena Provenzano, Hamid Raza Ali, Alexander Gaber, Gillian O'Hurley, Sophie Lehn, Jettie J.F. Muris, Jelle Wesseling, Elaine Kay, Stephen John Sammut, Helen A. Bardwell, Aurélie S. Barbet, Floriane Bard, Caroline Lecerf, Darran P. O'Connor, Daniël J. Vis, Cyril H. Benes, Ultan McDermott, Mathew J. Garnett, Iris M. Simon, Karin Jirström, Thierry Dubois, Sabine Linn, William M. Gallagher, Lodewyk F.A. Wessels[#], Carlos Caldas[#] & Rene Bernards[#]

[*] Contributed equally

# Corresponding author

Additional data files....4
Extended material and methods....4
Study design....4
Survival analysis....4
Affymetrix SNP 6.0 arrays....5
DNA Capture Library and Next-Generation Sequencing....5
RNA sequencing....6
Microarray Hybridization....7
Gene expression normalization and clustering....8
Reverse Phase Protein Arrays....8
Drug sensitivity....9
OncoScape....9
Gene expression and RPPA integration....10
Gene expression subtype pathway analysis....10
Decision tree....10
Supplementary figures....12
Fig S1. Venn diagram of the number of samples profiled on each platform....12
Fig S2. Tumour characterization....13
Fig S3. Gene expression clustering....14
Fig S4. CD4 and CD8 staining....14
Fig S5. Validation strategy....15
Fig S6. Validation in METABRIC and TCGA....16
Fig S7. Enrichment Maps....16
Fig S8. Subtype biomarkers in METABRIC....19
Fig S9. Gene signature enrichments....20
Fig S10. Mutational landscape....21
Fig S11. CDH1 expression....21

Fig S12. PI3K mutations 22

Fig S13. Recurrent CNAs segments 23

Fig S14. OncoScape candidate drivers 24

Fig S15. Survival analysis of the IR and HR subtypes 25

Fig S16. Cell lines 26

Fig S17. Differential drug response 27

Fig S18. Survival analysis of the mutation rate 28

Fig S19. Survival analysis of proteins 28

Fig S20. Decision tree performance assessment 29

Fig S21. Decision tree with clinical variables 30

Fig S22. Possible treatment effect 30

Fig S23. Subtype biomarkers and lymphocytic infiltration 31

Fig S24. PD-L1 immunohistochemitry 33

Fig S25. RPPA clustering 34

Supplementary Tables 34

Table S1. Integrative clusters on RATHER 34

Table S2. Lymphocytic infiltration 35

Table S3. IR/HR and TCGA subtypes 35

Table S4. Intrinsic and integrative clusters on METABRIC 35

Table S5. Systematically comparing IR and HR subtypes 36

Table S6. Adjusted Hazard ratios for known prognostic factors 37

Table S7. High cellularity clustering 37

References 38

ADDITIONAL DATA FILES

Additional file 1: Patient Table. Distribution summary of the quantitative and qualitative clinical variables characterizing each sample of the cohort.

Additional file 2: Kinome target genes. List of the genes targeted in the DNA sequencing experiments.

Additional file 3: RPPA epitopes. List of the epitopes targeting proteins and phospho-proteins in the RPPA experiments.

Additional file 4: GSEA results. Results of GSEA analyses using the gene signatures and the pathways for the RATHER and METABRIC datasets.

Additional file 5: Mutated genes. List all genes with candidate somatic variants found in any sample.

Additional file 6: Recurrent CNA. Multi-level recurrent CNA identified by ADMIRE.

Additional file 7: OncoScape. Prioritization scores of each gene tested using mutation, CNA, gene expression and RPPA data.

Additional file 8: Differential drug response. Results of the differential analysis for 88 drugs comparing the cell lines response in the HR and IR subtypes.

Additional file 9: RPPA survival. Results of the survival analysis for the proteins and phospho-proteins.

Additional file 10: METABRIC and RATHER-samples. List of samples part of both RATHER and METABRIC study.

Additional file 11: Factors components. Weights of the input contributing to each factor.

EXTENDED MATERIAL AND METHODS

STUDY DESIGN

All patients with an ILC (based on pathology report) treated in the NKI-AVL since 1980 were extracted from the hospital database. We excluded all patients for which no fresh frozen (FF) tissue was available in the NKI-AVL tissue bank. We selected consecutive tumours without neo-adjuvant treatment and with a preference for those also without adjuvant hormonal therapy. All patients diagnosed with ILC (based on pathology report) treated in the Addenbrookes Hospital Cambridge UK since 1997 and with available FF material were included in this study. Clinical data were extracted from the Addenbrookes Hospital Cambridge database. In some cases, we also sourced FF tissue from adjacent matched normal tissues. Subsequently, we collected matched formalin fixed paraffin embedded (FFPE) tissue blocks for TMA construction. The NKI-AVL and Cambridge medical ethical committees approved the study and the use of anonymized archival tissue in this study. The cohort consists of 144 samples.

SURVIVAL ANALYSIS

Since our survival analysis focused on associations with biological characteristics of the tumour, we excluded for survival analysis those patients that had another cancer diagnosis within 10 years before the diagnosis of ILC since it would be unclear to which tumour the event belongs. We considered only breast cancer specific survival, due to the presence of competing events and (distant) recurrence free survival. To plot patient stratification, we used Kaplan-Meier survival curves. P-values reported in

these figures are calculated with the log-rank test on the Kaplan-Meier estimator. All associations with survival were tested in Cox models including clinical parameters: Cox proportional hazards regression model was stratified by biobank and, unless otherwise specified, fitted with commonly used clinical variables: tumour size, grade, number of positive lymph nodes, treatment (hormonal, radiotherapy and/or adjuvant chemotherapy) and age at diagnosis. Association of a variable with survival was tested with a likelihood-ratio test comparing a model including clinical variables over a model including clinical variables and the variable tested. To assess the association of the EMT factor with survival in different datasets, we used the first principal component of the probes targeting the two major genes reported by Anastasiou *et al.*[1]: *COL11A1* and *THBS2*. Association of this first principal component with survival was tested in a Cox-model as described above. In the METABRIC dataset, we looked at breast cancer specific survival over the following clinical variables: grade, size, stage, number of positive lymph nodes, age at diagnosis and subtype. Luminal samples were selected based on having a PAM50 annotation, as provided by METABRIC, of Luminal A or B.

AFFYMETRIX SNP 6.0 ARRAYS

The protocol was as presented earlier[2]. Briefly, DNA was extracted from ten 30 μm sections each from fresh frozen tumours using the DNeasy Blood and Tissue Kit and the miRNeasy Kit (Qiagen, Crawley, UK) on the QIAcube (Qiagen) according to the manufacturer's instructions and then hybridized to Affymetrix SNP 6.0 arrays per the manufacturer's instructions (Affymetrix, Santa Clara, CA) at AROS Applied Biotechnology (Aarhus, Denmark).

Each sample was preprocessed using the PennCNV pipeline for Affymetrix arrays[3]. Genotyping calls were obtained with Affymetrix Power Tools (APT) software using the Birdseed algorithm (http://www.affymetrix.com/partners_programs/programs/developer/tools/powertools.affx). Allele-specific signals were extracted and a canonical genotype clustering file was generated using all samples. Each array was then wave-corrected using the built-in algorithm in ASCAT v.2.2[4] and copy numbers were called with ASCAT v2.2 using information from the matched normal when available. The samples were classified into the 10 integrative clusters from METABRIC using the iC10 package with default parameters and the "scale" normalization method[5]. We used copy number data (segmented means obtained with DNAcopy) and expression data from the Agilent arrays. The overall goodness of fit of the correlation was 0.772.

Ploidy of samples was estimated by ASCAT. We found two clusters of samples with similar ploidy: one cluster with ploidy around two, and one cluster with ploidy around 3.8 (Figure S23). Therefore we consider the samples with an ASCAT estimated ploidy higher than 3 as tetraploid. If a sample is tetraploid, the expected copy number is four, so we want to consider regions with a copy number of four as being copy number neutral. Copy number of all segments in the tetraploid samples was divided by two prior to the employing ADMIRE to find recurrent aberrations. We then applied ADMIRE [6] to identify recurrent alterations, clipping at a CN level of 6 and with an FDR threshold of 0.05. For all recurrences found, a CN was calculated per sample by taking the median CN of all segments overlapping a recurrence. Differential copy number between the subtypes was determined by a Wilcoxon permutation test (R coin package) because of the large number of ties. When both a focal copy number difference and a larger overlapping copy number difference were found, we only report the larger one.

DNA CAPTURE LIBRARY AND NEXT-GENERATION SEQUENCING

DNA sequencing was performed on an Illumina HiSeq 2000 platform. For each sample, Illumina TruSeq index libraries were constructed according to manufacturer's instructions (Illumina) before being enriched by capture with a biotinylated RNA probe set targeting the human kinome and a range of cancer related genes (Agilent Technologies, 3.2 Mb). We sequenced 10 to 12 samples on a single Illumina HiSeq 2000 lane to generate 55bp paired-end reads. On average, we obtained 26,985,771

unique reads on each run. The average kinome coverage (mean bait coverage) for the whole sequencing dataset is 133X, ranging from 36 to 258. On average, 91% of the target positions are covered by 20x. We aligned the raw sequencing data with the Burrows-Wheeler Aligner (BWA) version 5.10, backtrack algorithm, to the human genome (Ensembl 37) removing duplicate reads and reads with mapping quality <60. We used SAMtools mpileup to identify variants found in the targeted region +/- 100bp. We then employed the vcfutils.pl script provided with SAMTools to filter variants using the varFilter set to defaults with the exception of turning off the -2 float option. We called the subsequent variants using SAMTools and followed the following filtering process: we kept only variants matching the following criteria: i) root-mean-squared mapping quality MQ>40 ii) variant frequency > 0.1 iii) total coverage > 10 iv) variant coverage > 5 v) fraction of reads with the alternative allele occurring in one read direction > 7.5 % of the reads for the given direction (to avoid strand bias). 15806 variants passed these criteria. We kept only variants predicted to alter the proteins using the Ensembl variant effect predictor (VEP) and the following categories: missense variant, stop gained, frameshift variant, inframe insertion, inframe deletion, splice donor variant, splice acceptor variant, initiator codon variant (i.e. removing synonymous variants). 3122 variants remained, which we classified into 2169 germline and 953 somatic, based on the following rules: 1) if a variant is present in at least one of the normal samples of our in-house pool, it is considered germline; 2) if a variant is reported in a database (dbSNP, Exome Variant Server esp5400 database) and it is not present in COSMIC, it is considered germline; 3) otherwise, it is considered somatic. To assess the significance of the mutations in a gene, we compared its mutation frequency to the average background mutation rate taken into account its size and using a Binomial test (Mutascape package). All p-values were then adjusted for multiple testing with a Benjamini-Hochberg correction.

A set of candidate somatic variants were selected for validation by sequencing tumour and matched normal material, which was extracted from FFPE lymph nodes that were free of tumour cells, or from adjacent non-involved breast tissue. These samples were analysed with kinome capture sequencing (n=92), traditional capillary sequencing, or with small PCR amplicons targeting the variant that were pooled for Illumina based sequencing. Variants found back in the tumour sample and not in the normal are validated mutations (VALIDATED); variants found in both the tumour and the normal samples are rare germline variants (SNP); variants not found back in the tumour samples are false positive calls (ABSENT); finally some variants were tested but the experiment failed (FAILED).

	VALIDATED	SNP	ABSENT	FAILED	TOTAL
Counts	199	282	9	5	495
%	40	57	2	1	100

We note that these numbers relate to variants, but not to their frequency in our dataset. Thus, if frequent variants are validated as somatic mutations (as is the case for PIK3CA hotspot mutations), the overall percentage of true somatic mutations in the dataset is much higher than in the table.

Recently, Ross et al. reported a high frequency (30%) of ERBB2 mutations in ILC as compared to overall breast cancer (5%)[7]. Approximately 7% of ILCs in our cohort had mutations in ERBB2. Even when restricting our analysis to only CDH1-mutated tumours, as was the case in Ross et al., we still have a low mutation frequency (4%). These differences may be due to the fact that we have sequenced DNA derived from primary cancers at diagnosis, whereas Ross et al. focused on biopsies from progressive disease. One possible explanation for this difference is that ERBB2 mutations are selected for during disease progression in ILC.

RNA SEQUENCING

RNA-sequencing data were used to specifically estimate the absolute expression levels of *CD4*, *CD8A* and *CD19* in both subtypes. RNA sequencing was performed on a subset of 68 ILC clinical samples. The sequencing was carried out by BGI, Hong Kong, using their stand-specific paired-end transcriptome sequencing pipeline. Briefly, for each sample, oligo(dT) magnetic beads were used to isolate poly(A) mRNA from the total RNA preparation. A fragmentation buffer was used to cleave the mRNA into short fragments. Random hexamer primers were used to synthesize the first cDNA strand from these template fragments. dNTPs were removed, and the second-strand cDNA was synthesized using buffer, dATP, dGTP, dCTP, dUTP, RNase H and DNA polymerase I, respectively. Short fragments were purified with the QiaQuick PCR extraction kit, and resuspended in EB buffer for end-repair and poly(A) addition. Next, the short fragments were ligated with sequencing adaptors. Uracil-N-glycosylase (UNG) was used to digest the second cDNA strand. cDNA was size-selected using an agarose gel (~200bp insert size) and subjected to PCR amplification to complete the sequencing library.

Paired-end sequencing was carried out for 90 cycles on an Illumina HiSeq 2000 platform. The raw data was filtered to remove low-quality reads and reads containing adaptor sequences. Following this step, approximately 50 million 90bp 'clean' read pairs were available for each sample. Quality was subsequently assessed using FASTQC v0.10.1 (Andrews, 2010). Reads that mapped to ribosomal RNA or mitochondrial sequences were removed from subsequent analysis. The remaining read pairs were aligned to the GRCh37 genome with TopHat v2.0.10[8], using Bowtie 2.1.0 as the underlying aligner. Reads aligning to Ensembl 75 genes were quantified with featureCounts[9], which discounted any read pair that aligned to more than one location, or more than one gene at a single location. DESeq2[10] was used to normalize the read counts and derive FPKM values. For the purposes of the FPKM calculation, the length of a gene was defined as the number of base pairs covered by any transcript of that gene.

To determine if the IR and HR subtypes identified by microarray gene expression clustering are supported by RNA-seq data, a clustering approach similar to the one for the microarray gene expression, described in the next section, was adopted. DESeq2 was used to derive regularized log transformed read counts. Genes were ranked by median absolute deviation (MAD; calculated by R using the default scaling factor), and the 1000 genes with the highest values were selected for clustering. Each gene's values were standardized using the gene's mean and standard deviation. Values were capped at +/- 2 standard deviations. ConsensusClusterPlus[11] was used to cluster the samples into two clusters, using the same key parameters used for calculating the microarray consensus matrix (10,000 repetitions, average linkage, Pearson distance, 90% gene resampling). The resulting consensus matrix was hierarchically clustered using average linkage, and all samples were assigned to a cluster. To compare gene expression for immune-related genes between the IR and HR clusters, DESeq2 was used to perform a differential gene expression analysis.

Microarray Hybridization

The RNA for microarray analysis and sequencing was purified using the Qiagen RNeasy micro kit (Qiagen, Hilden, Germany) according to manufacture's protocols. Shortly, tumour samples were thawed at 37°C (±3°C), put on ice, and homogenized with a polytron and centrifuged. The supernatant was transferred to a new tube, 100 ml CHCl3 added and centrifuged again. 250 ml of water phase were transferred to a new tube and 350 ml 70% EtOH added. After vortexing, 500 µL were transferred to an RNeasy column, washed with RW1 buffer, treated with DNase and eluted with water. RNA concentration was determined with Nanodrop and RNA quality with the Bioanalyzer. Samples with RIN above 5 (2100 Bioanalyzer, Agilent Technologies) were selected for further analysis. RNA was amplified, labelled and hybridized to the Agendia custom-designed whole genome microarrays (Agilent Technologies) and raw fluorescence intensities were quantified using Feature Extraction software (Agilent Technologies) according to the manufacturer's protocols. We checked the quality of the array printing, background noise, intensity and array uniformity using a series of 250 control probes. In addition, each step of the process of RNA isolation, amplification and expression analysis

uses instruments and quality measurements described and developed for the FDA-cleared MammaPrint analysis process[12].

Gene expression normalization and clustering

Feature signal intensities were processed and extracted according to the limma Bioconductor R package with background subtraction using an offset of 10. All probe intensities <1 were set as missing values. These missing values were imputed by 10-nearest neighbor imputation (R-package impute) prior to analysis that cannot deal with them. The log2 transformed probe intensities were quantile normalized[13] using limma. A principal component analysis showed a batch effect for biobank, and an additional batch of samples was identified that were cut at the same time (identifiers RL1110–RL1130). Both batch-effects were adjusted for using ComBat[14]. Genes with multiple probes were summarized by the first principal component of a correlating subset (all probes with correlation to any other probe >0.5), if such a subset existed or by the most variable probe if no such subset existed. After summarizing by first principal component, signs and variance were adjusted to match with the most variable probe of a gene. Some genes (43) showed a discordant signal over multiple probes, so were not summarized and thus kept as separate probes.

We applied several different clustering algorithms on the top 1000 genes with highest median absolute deviance: hierarchical clustering with Pearson distance and ward D1, single, average and complete linkage, as well as non-negative matrix factorization (NMF). The ward D1, average and NMF methods gave stable clustering results as assessed by consensus clustering. When choosing two clusters, all three methods found largely the same two clusters (Figure S3). To define subtypes, we first performed consensus clustering with average linkage, two clusters, and 90% feature resampling. Then, the consensus matrix was hierarchically clustered with complete linkage and Euclidean distance. Finally, the resulting tree was cut at a quarter of maximum height, defining two big clusters. Samples not falling into one of these two clusters were not assigned to any cluster (n=42). NMF was done with the R package NMF, consensus clustering with the ConsensusClusterPlus package[11]. To assign cell lines to clusters, we normalized together the raw gene expression data of cell lines and tumour samples. Then, we applied the same clustering approach described above, but cut the tree at maximum height such that all cell lines where assigned to a cluster. All tumour samples assigned to a cluster were assigned to the same cluster in both clustering results with and without cell lines.

Reverse Phase Protein Arrays

Three sections of fresh frozen tissue were lysed in hot Laemmli buffer (50 mM Tris pH 6.8, 2% SDS, 5% glycerol, 2 mM DTT, 2.5 mM EDTA, 2.5 mM EGTA, 1x HALT Phosphatase inhibitor (Perbio 78420), Protease inhibitor cocktail complete MINI EDTA-free (Roche 1836170, 1 tablet/10 mL), 2 mM Na_3VO_4 and 10 mM NaF) and boiled for 10 min at 100°C. Samples were sonicated in a waterbath for 1-2min to break the DNA and centrifuged for 10 min at 13000 rpm. Supernatant was snapfrozen and protein concentration was measured (BCA reducing agents compatible kit, Pierce, Ref 23252). Samples with sufficient protein concentration (>0.5 mg/ml) were deposited onto nitrocellulose covered slides (Sartorius, Grace Biolabs or Maine Manufacturing) using a dedicated arrayer (2470 Arrayer, Aushon Biosystems). Five serial dilutions, ranging from 0.5 to 0.03125 mg/ml, and two technical replicates per dilution were deposited for each sample. Arrays were labelled with commercially available antibodies using an Autostainer Plus robot (Dako). Briefly, slides were incubated with avidin, biotin and peroxydase blocking reagents (Dako) before saturation with TBS containing 0.1% Tween-20 and 5% BSA (TBST-BSA). Slides were then probed overnight at 4°C with primary antibodies diluted in TBST-BSA. After washes with TBST, arrays were probed with horseradish peroxidase-coupled secondary antibodies (Jackson ImmunoResearch Laboratories, Newmarket, UK) diluted in TBST-BSA for 1 h at RT. To amplify the signal, slides were incubated with Bio-Rad Amplification Reagent for 15 min at RT. The arrays were washed with TBST, probed with Cy5-Streptavidin (Jackson ImmunoResearch Laboratories) diluted in TBST-BSA for 1 h at RT and washed again in TBST. For

staining of total protein, arrays were incubated 15 min in 7% acetic acid and 10% methanol, rinsed twice in water, incubated 5 min in Sypro Ruby (Invitrogen) and rinsed again. The processed slides were dried by centrifugation and scanned using a GenePix 4000B microarray scanner (Molecular Devices). Spot intensity was determined with MicroVigene software (VigeneTech Inc). Specificity of each primary antibody used in this study was first validated by Western blotting on a panel of cell line lysates representative of human tumours of diverse origins. For each sample, one relative protein expression level was determined from the technical replicates and the dilution series, using Normacurve software[15]. Normacurve takes into account all samples on the array to draw a robust antibody response curve. Next, for each sample, the individual dilution curve is fitted onto this antibody response curve and the median expression level is read from the curve. In addition, Normacurve applies a spot-by-spot normalization for background fluorescence (using a slide incubated without primary antibody), for total deposited protein (using a slide labelled with a total protein stain) and for potential spatial bias on the slide[15]. Bias due to origin of the samples (NKI vs CAM) was removed using a median regression approach. In brief, data were scaled by array and the median for each sample across all arrays was computed. Then, linear regression was performed of scaled data on the median of proteins and residues were set as the final processed data.

Hierarchical clustering was applied to the RPPA data in order to classify the ILC samples, using the Pearson metric and Ward agglomerative method. Four clusters were retained based on the results of Silhouette, Davies-Bouldin index and consensus clustering. Enrichment of HR and IR subtypes in the RPPA clusters was analyzed by chi-square distribution test. Differentially expressed proteins between the clusters were identified using linear models and analysis of variance.

At the protein level, we identified four clusters, which clearly show different patterns of cell signalling (Figure S25). We found that the RPPA clusters showed a non-even distribution in the HR subtype ($p=0.002$), with enrichment in RPPA cluster 4 ($p=0.023$). RPPA cluster 4 contains 44% (12/27) of all HR samples against 25% expected by chance. RPPA cluster 4 over-expresses proteins involved in HR signalling, such as P-ERα-Ser118 ($p=0.0074$), GATA3 ($p=0.0012$) and 4EBP1 ($p<10e-6$), compared to the three other clusters. In contrast, the IR subtype does not show overlap with a particular RPPA cluster, possibly because the proteins characterizing this cluster (cytokines and other immune-related genes) have not been measured by RPPA.

Drug sensitivity

We profiled a panel of 15 cell lines identified as ILC-like based on genetic criterion: cell lines have either a *CDH1* or a-catenin genetic event associated with loss-of-function and are therefore deficient in the complex that we consider to be a hallmark of lobular cancers. Drug sensitivity was assessed on the Sanger cell line panel (internal version 17). We used the cell lines common in our ILC cell line panel and in the Sanger cell line panel. We used cell line AU565 instead of SK-BR-3, which is derived from the same patient. Among the 262 drugs, we focused our assessment on 88 agents that had measurement in at least three cell lines per subtype. With this dataset, we performed a two-sided t-test between the AUC of the dose-response curves of the cell lines in the two subtypes, correcting for multiple testing with the Benjamini-Hochberg method. We show the IC50 in the figure for easier interpretation.

OncoScape

We used OncoScape for comparing gene expression, RPPA protein expression, copy number alteration and mutations between IR and HR subtypes. Each of these categories is analyzed separately by OncoScape. For the first three data types, we compared numerical values for each gene between the two subtypes using the Wilcoxon test and defined genes as significantly different if the Benjamini-Hochberg corrected p-value was < 0.05. For copy number data, OncoScape additionally required that the copy number values were significantly correlated (Benjamini-Hochberg corrected p-value < 0.05) with gene expression. Upregulation and copy number gains were defined as oncogene-like aberrations

while downregulation and copy number losses were defined as tumour suppressor-like aberrations. Mutations were analyzed according to the 20/20 rule defined by Vogelstein *et al.*[16]. For this analysis, missense variants, coding sequence variants and inframe indels were considered as possible oncogene mutations, while truncating, frameshift and splice region mutations were considered as potential tumour suppressor gene mutations. Also, we required at least five oncogene or tumour suppressor mutations for individual genes in order to avoid spurious calls. If a gene was found to be altered in one of the four data types mentioned above, it received a score of 1 for this data type and else a score of 0. Summing up all oncogene-like aberrations yielded the oncogene score and the sum of all tumour suppressor-like aberrations resulted in a tumour suppressor score, respectively. All four categories were weighted equally for calculating oncogene and tumour suppressor scores. Additionally, we calculated the difference between oncogene score and tumour suppressor gene score and referred to it as overall score. We included all genes with available gene expression, RPPA protein expression and copy number data in the OncoScape analysis.

Gene expression and RPPA integration

We first applied a factorization integrating RPPA and gene expression data, and then did a pathway analysis on these factors within the gene expression data. To extract concordant data for the factorization, we selected only the expression of the 1391 genes that were in the top 10 correlating (absolute Pearson's ρ) with any RPPA epitope. All RPPA epitopes were used. The iCluster method[17] was re-purposed to perform factorization, by foregoing the k-means clustering step at the end. Also, uniform sampling was used to select shrinkage parameters and number of factors resulting in the highest proportion of deviance. The weights of the features for each factor are provided in Additional file 13. We adapted gene set enrichment analysis (GSEA)[18] to perform a pathway analysis on the factors. We constructed ranked lists of genes per factor by regressing the factors on expression data of all genes and then scaling the regression coefficients of a gene by its variance. Overrepresentation of a pathway on top of a list was calculated with the weighted GSEA score. Significance was assessed by sample permutation. For pathway analyses we used GSEA with the mSigDB v4.0 'canonical pathways' (called pathways) and 'chemical and genetic perturbations' (called signatures) gene set collections. In this analysis, the gene expression signature defined by Anastassiou *et al.*[1] came as significantly associated with one of the factors, leading us to the EMT interpretation of that factor.

Gene expression subtype pathway analysis

To contrast both IR and HR subtypes we also used GSEA with the mSigDB v4.0 'canonical pathways' (pathways) and 'chemical and genetic perturbations' (signatures) gene set collections. Genes were ranked by differential expression (signal-to-noise ratio) between the two clusters. To investigate more specifically oestrogen signalling, we used the list of up and down regulated genes upon oestrogen stimulation of MCF-7 cells as determined by Zwart *et al.*[19]. Up or down regulation of these genes between the HR subtype, as compared to the IR subtype, was assessed with one-sided Wilcoxon ranked-sum tests and a p-value cutoff of 0.05. 451 of 987 up-regulated genes were also up-regulated in the HR subtype and 234 of 915 down-regulated genes were also down-regulated in the HR subtype, significantly more than expected in both directions (binomial test, $p<1e-6$). The gene expression signature that recapitulates the EMT factor is specific for EMT and not fibroblast as investigated by the authors in a mouse xenograft model[1].

Decision tree

Decision trees were built using conditional inference trees[20]. We used the implementation in the R package party. We applied Bonferroni correction, used a p-value threshold of 0.25, a minimum of 20 samples to split, and a minimum of 10 samples in a leaf node. We wanted to combine high-level features and some that were associated with survival to try and get a robust and accurate predictive model together with easily to interpretable features. As high-level features, we considered i) mutation

rate and CNA rate (proportion of genome altered from the copy number data) as a summary for the level of genetic instability and ii) the EMT factor, which was the strongest component of the integrated analysis of gene expression and RPPA data. As features associated with survival, we used the epitopes from RPPA that showed a significant association with survival with a likelihood-ratio test. The thresholds we used to define the final tree are based on a tree trained with clinical variables as additional variables. Performance of the tree was assessed by partial likelihood deviance from a leave-one-out cross-validation. Four different models were tested, all including clinical features: i) the first model included only clinical features. ii) The second model also includes the epitopes from RPPA that showed a significant association with survival with a likelihood-ratio test. iii) The third model includes the clusters assignments from a tree trained on training data. iv) The fourth model includes the features used in a tree trained on training data. If the fitting procedure of a model would not converge, we used the model including only clinical features instead.

SUPPLEMENTARY FIGURES

FIG S1. VENN DIAGRAM OF THE NUMBER OF SAMPLES PROFILED ON EACH PLATFORM

We performed a comprehensive molecular profiling of 144 untreated tissue samples from primary ILC tumours. Specifically, we have used: (i) targeted DNA sequencing to study somatic variants on a set of 613 genes (**Mutations**); (ii) SNP6 arrays to study somatic copy number alteration (**CNA**) profiles; (iii) DNA microarrays to study gene expression (**GE**) and (iv) reverse-phase protein arrays (**RPPA**) to characterize the levels of a selected set of 168 proteins and phospho-proteins. We show here the number of samples successfully profiled on each platform for (A) the overall dataset and (B) the subset of samples assigned to one of the gene expression subtype described later on.

A All 144 samples

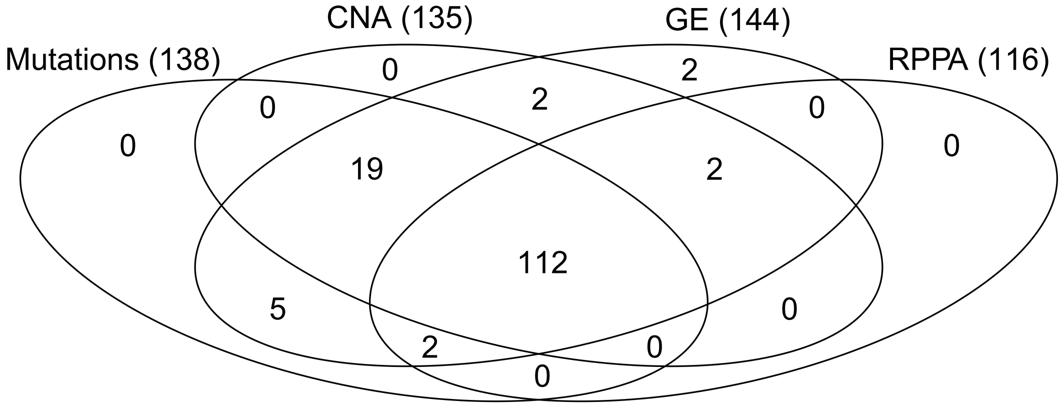

B 102 samples in IR and HR subtypes

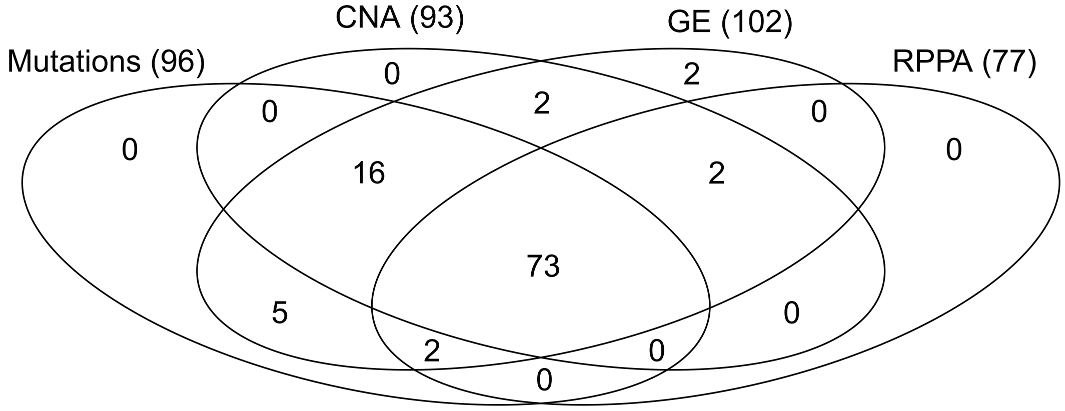

FIG S2. TUMOUR CHARACTERIZATION

We show here the ER fraction by immunohistochemistry (IHC) and the mRNA level of oestrogen receptor *ESR1*. Expression level of GATA3 is indicated by the colour scale (high level in red). Almost all samples are ER positive. Among the samples assessed as ER negative by IHC, the majority show ER mRNA expression. In fact, only a single sample seems to be triple negative (TNBC) (bottom left of the plot, showing low ESR1 and GATA3 expression). (B) ERBB2 expression split by ER/HER status by IHC. The TNBC sample is highlighted in red.

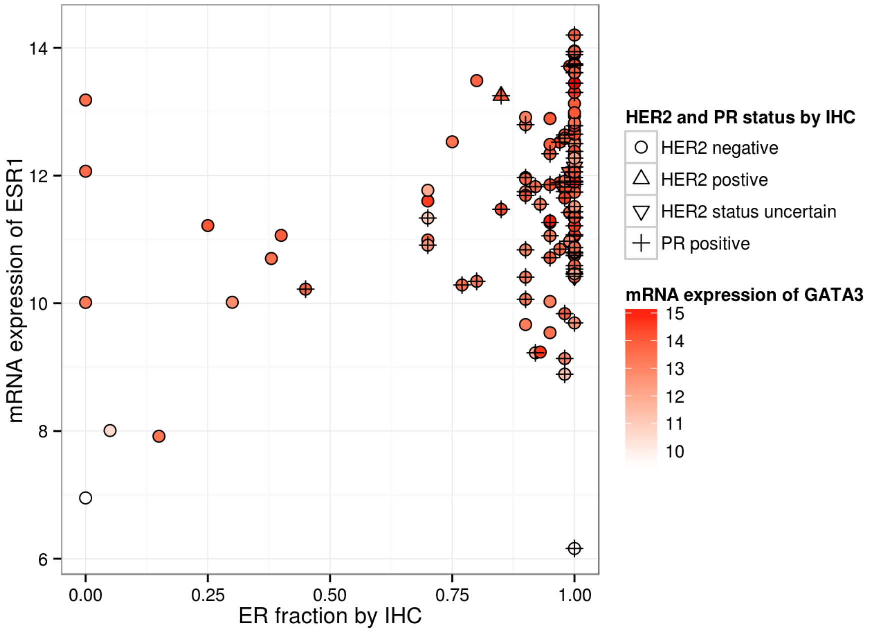

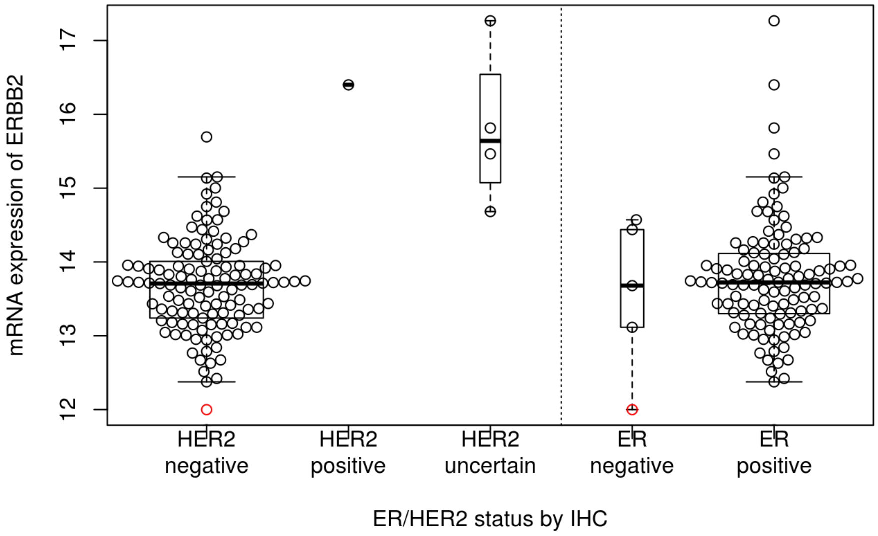

FIG S3. GENE EXPRESSION CLUSTERING

Gene expression clustering is very robust and different methods give highly overlapping results: hierarchical clustering with ward (**ward**) or average (**average**) aggregation criterion and non-negative matrix (**nmf**) factorization. Based on a gene sub-sampling analysis, we defined the final assignment (**consensus**): samples recurrently associated with the same subtype were assigned to it (IR in orange and HR in green), while samples changing subtypes were left unassigned (in grey).

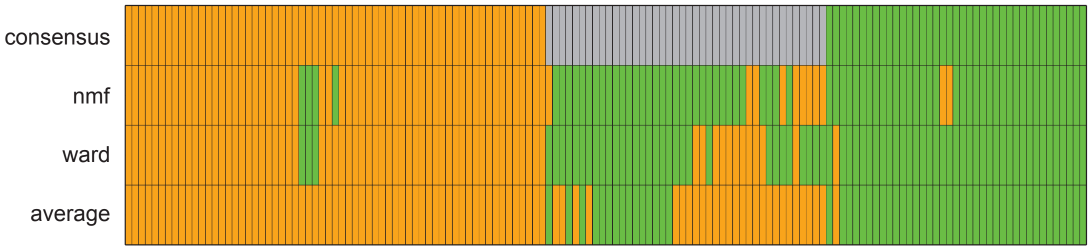

FIG S4. CD4 AND CD8 STAINING

We show here the number of cells staining positive for CD4 and CD8 expression. We compare the distributions of the samples in the IR and HR subtypes with a one-sided t-test. We also compared the log10 of the counts to stabilize the results (higher variance in IR for CD8). In all cases we observe a significant difference between the subtypes.

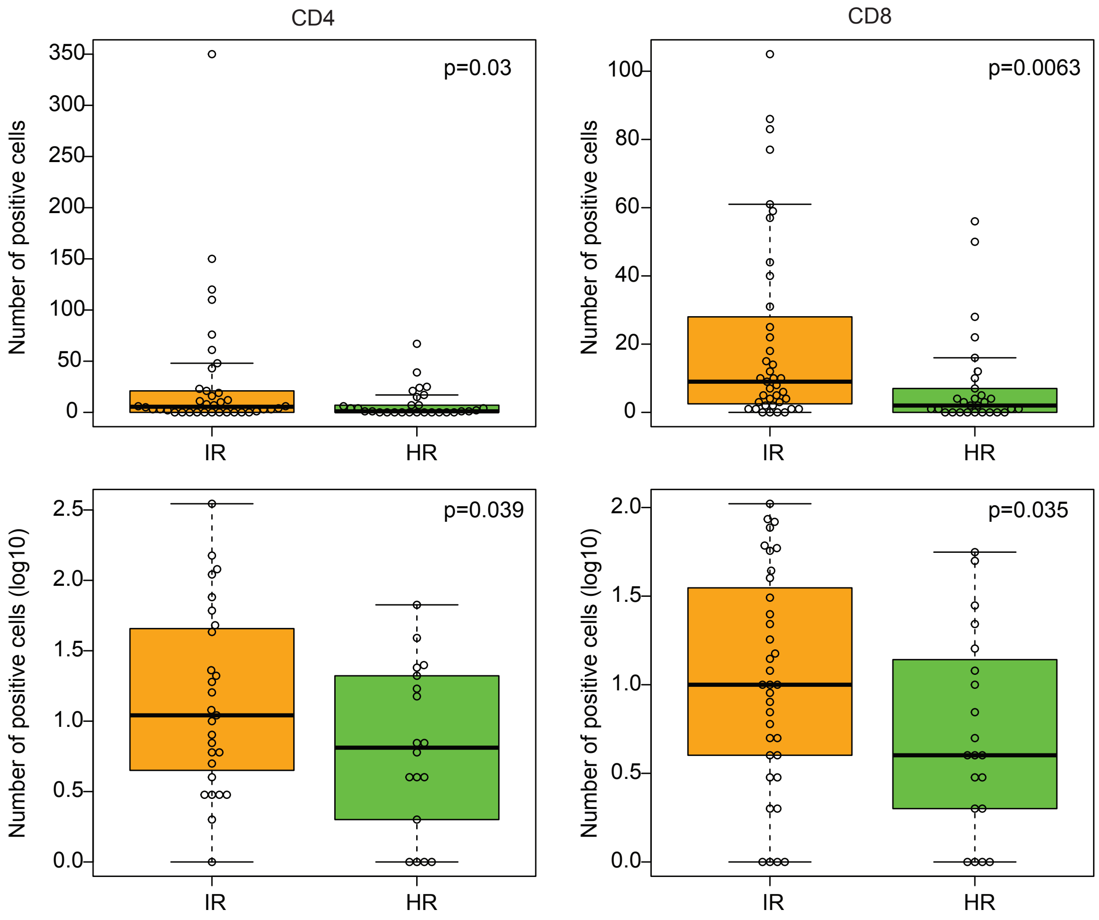

FIG S5. VALIDATION STRATEGY

To validate the IR and HR subtypes discovered on the RATHER dataset, we have used the same approach de novo on the ILC samples of two external validation datasets (METABRIC and TCGA): robust clustering, identification of two subtypes, identification of differentially expressed genes, pathway and signature enrichment analysis (as illustrated for METABRIC below).

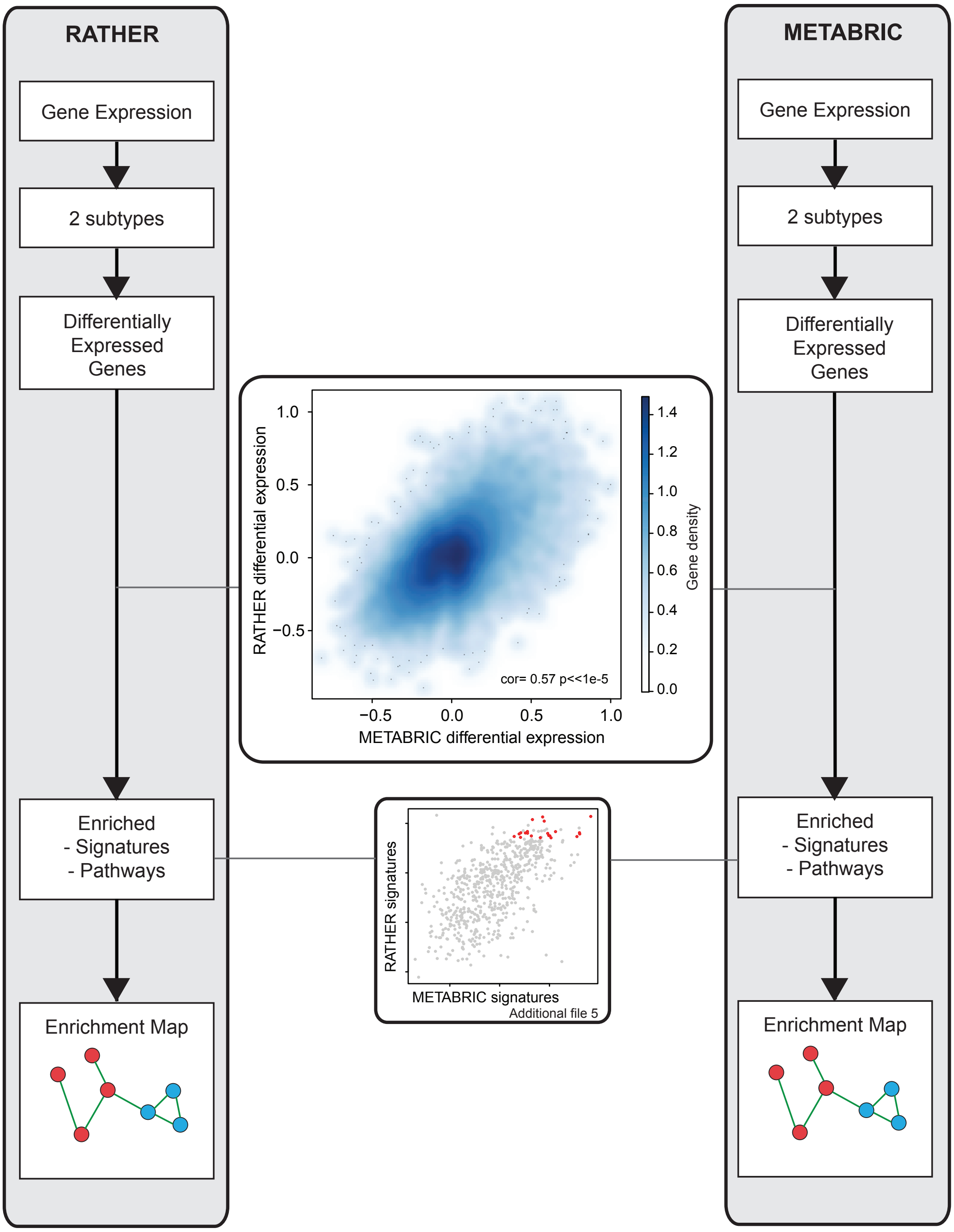

Fig S6. Validation in METABRIC and TCGA

The differential gene expression is highly correlated between RATHER and METABRIC and between RATHER and TCGA.

A. METABRIC

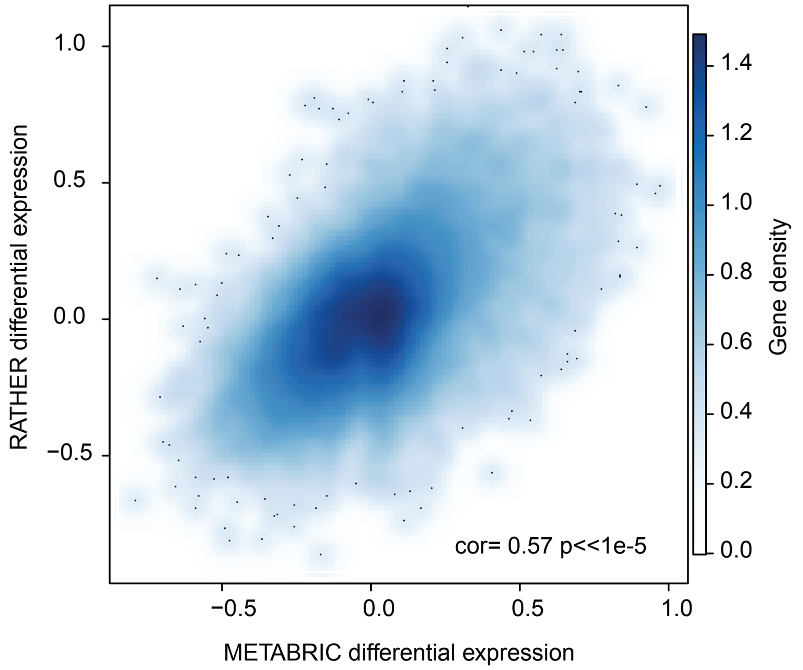

B. TCGA

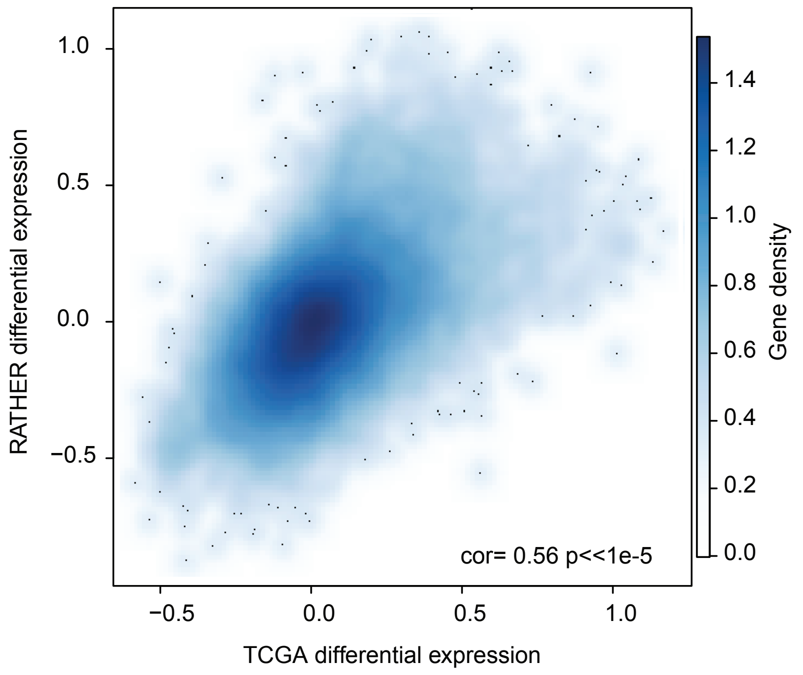

Fig S7. Enrichment Maps

Detailed Enrichment Maps for A) RATHER, B) METABRIC and C) TCGA.

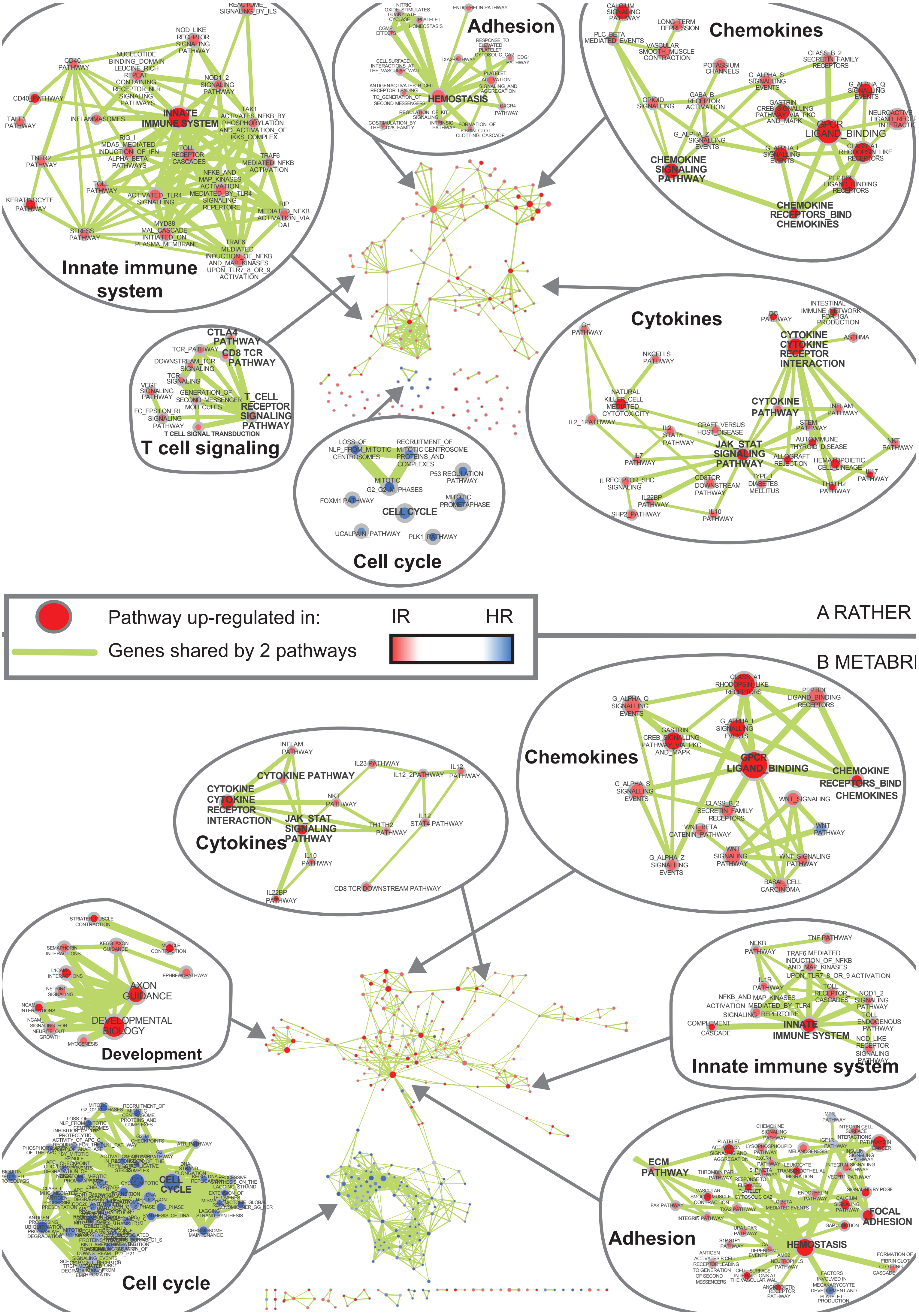

Innate immune system
Adhesion
Chemokines
T cell signaling
Cytokines
Cell cycle
Pathway up-regulated in:
Genes shared by 2 pathways
IR
HR
A RATHER
B METABRI
Chemokines
Cytokines
Development
Innate immune system
Cell cycle
Adhesion

Two subtypes of ILC

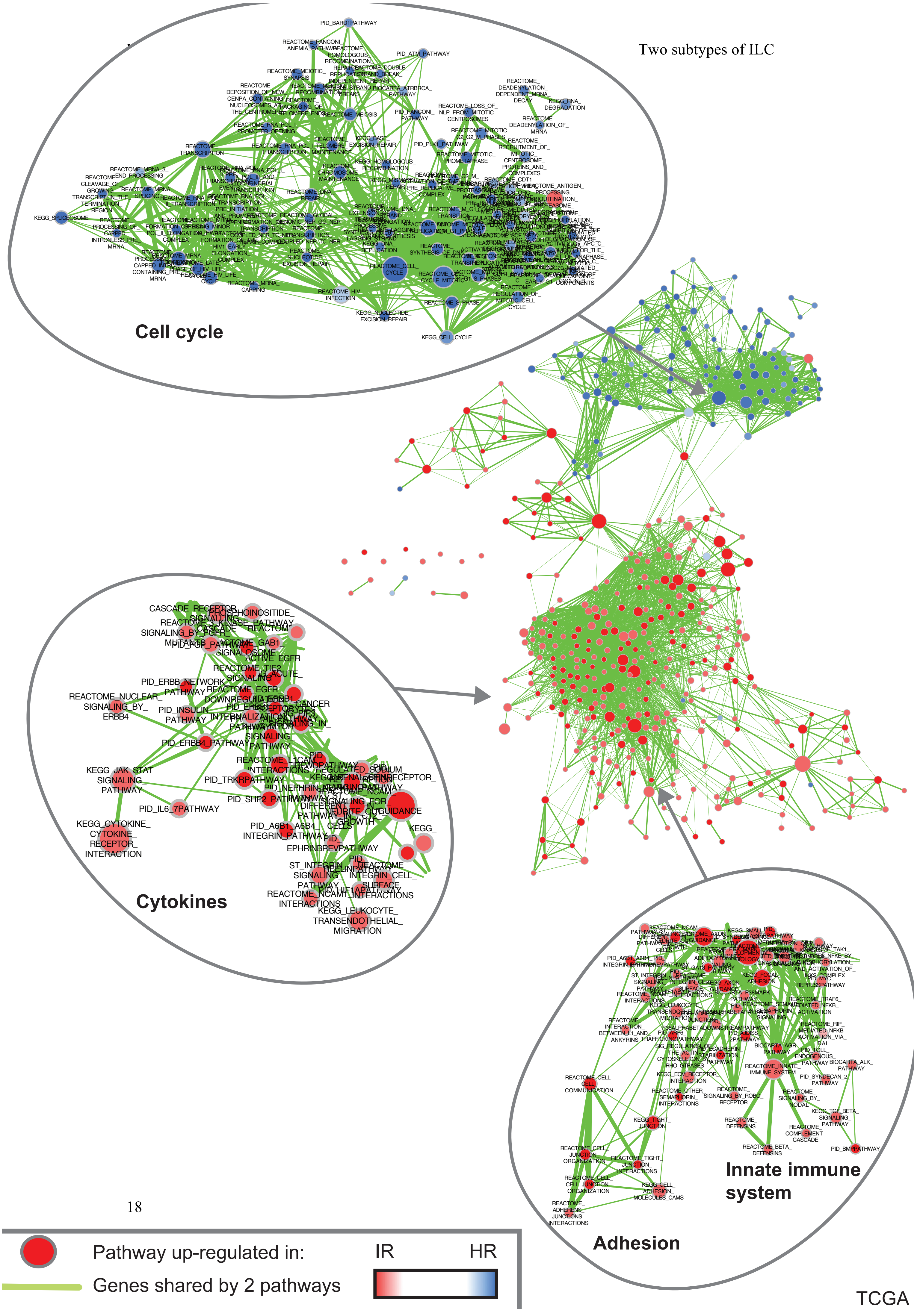

Fig S8. Subtype biomarkers in METABRIC

Each boxplot illustrate the gene expression of a given probe (e.g. ILMN 1806725) in a given gene (e.g. PDCD1) in samples of the IR and HR subtypes in the METABRIC validation dataset. We mapped probes to genes with the ReMOAT annotation[21]. We performed a Wilcoxon test and indicated the resulting p-value below each plot.

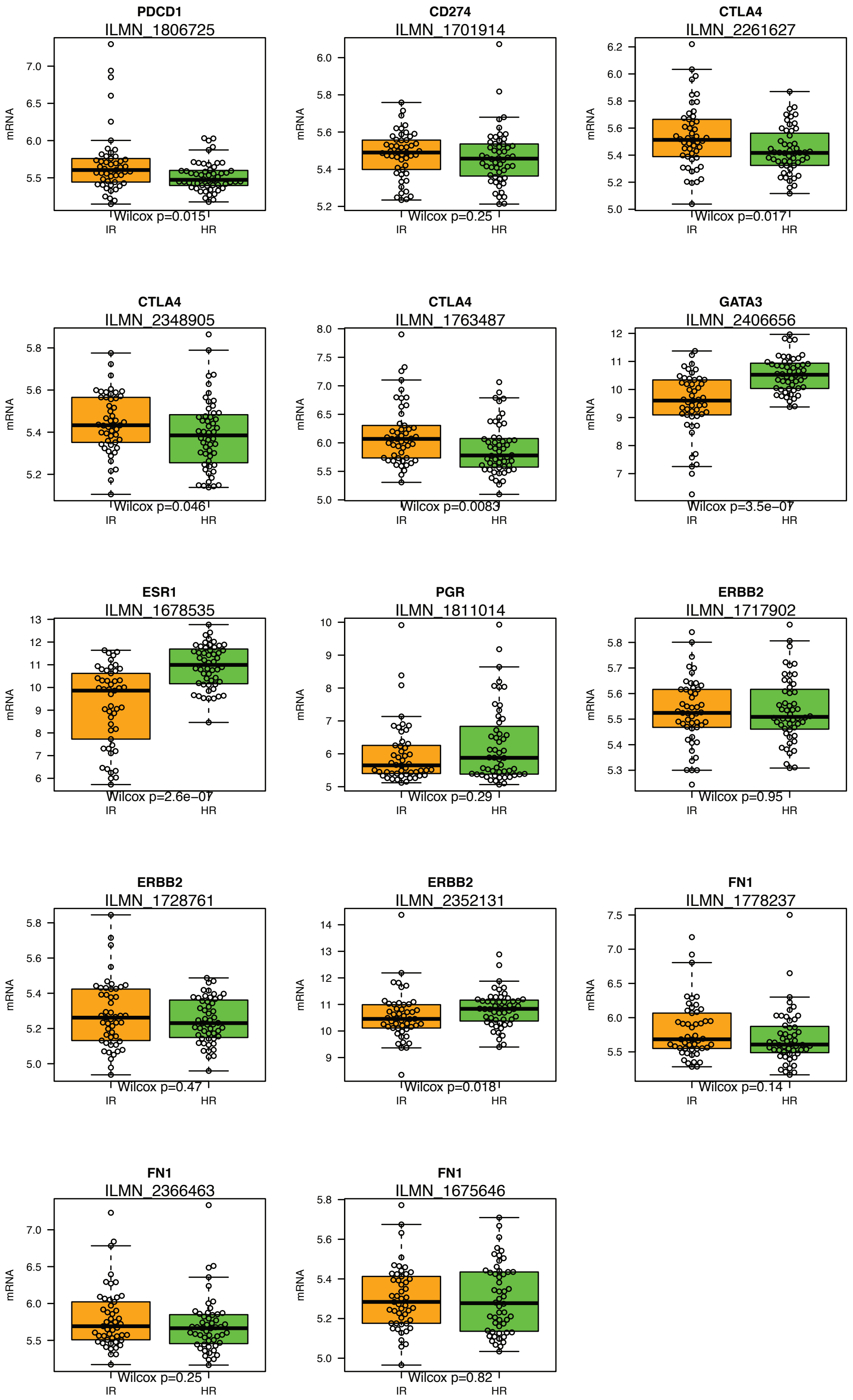

FIG S9. GENE SIGNATURE ENRICHMENTS

We show here the GSEA results on the gene signatures in RATHER and METABRIC for the A) IR and B) HR subtypes.

A Immune related (IR)

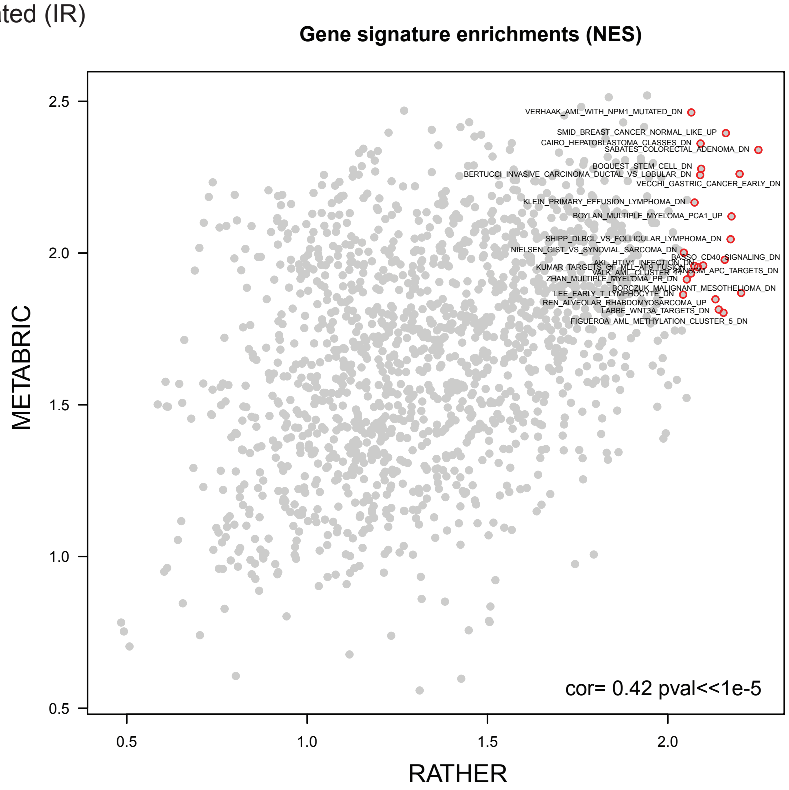

B Hormone related (HR)

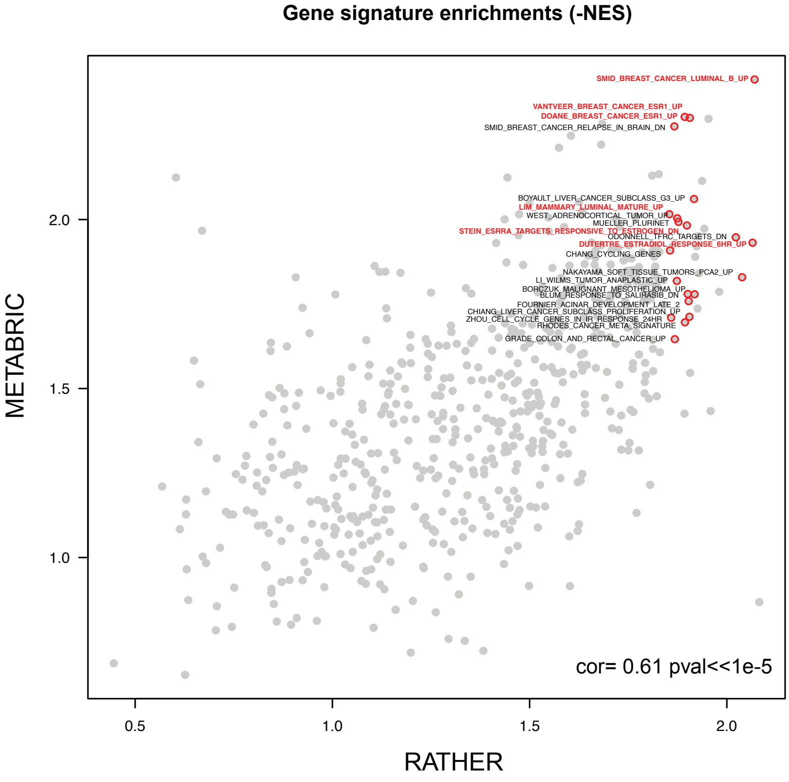

Fig S10. Mutational landscape

We show here a gene-centric view of the candidate somatic variants. (A) Nine genes are mutated in 5% or more of the samples. Three of them are significantly mutated with respect to their size (indicated in black). (B) We represent the mutational landscape with a bubble plot created with the Mutascape package (under development). Each gene is represented as a bubble, which center is positioned according to its size on the x-axis (Gene size in log scale) and its mutation frequency on the y-axis (% of samples mutated in the cohort). Bubble size indicates the statistical significance (FDR-adjusted p-values of a binomial test taking into account the gene size) and colour represents the type of mutation pattern, e.g. recurrent or non-recurrent (genes in red tend to have mutations at recurrent positions, while genes in white tend to have mutations at unique positions in the various samples).

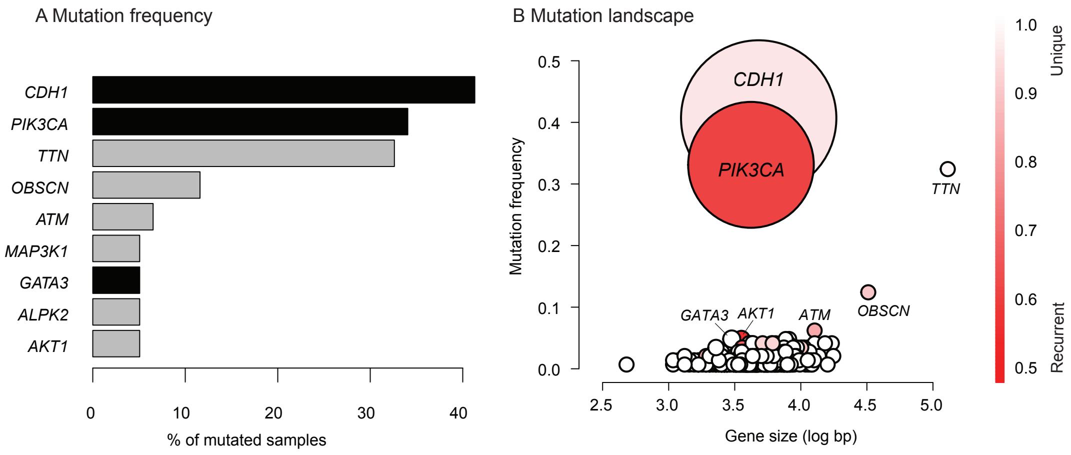

Fig S11. CDH1 expression

This scatter plot shows *CDH1* expression at mRNA (microarray) and protein (RPPA) levels for samples with and without somatic mutations in *CDH1*.

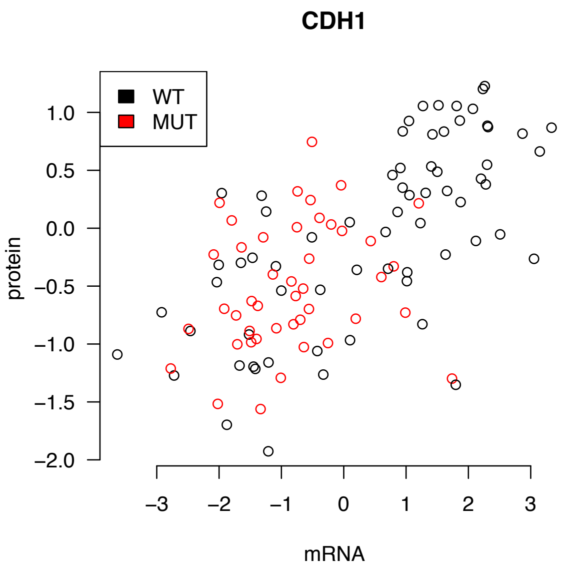

FIG S12. PI3K MUTATIONS

The heatmap shows the presence (in black) of mutations (and 1 loss in PTEN) in members of the PI3K pathway in all samples with DNA sequencing data. The PI3K pathway is mutated in 63 of the 138 tumours (46%) with mutations in *AKT1*, *PIK3R3*, *PTEN*, *PIK3CB*, *PIK3CG*, *PIK3CD* and *PIK3CA* that tend to be mutually exclusive.

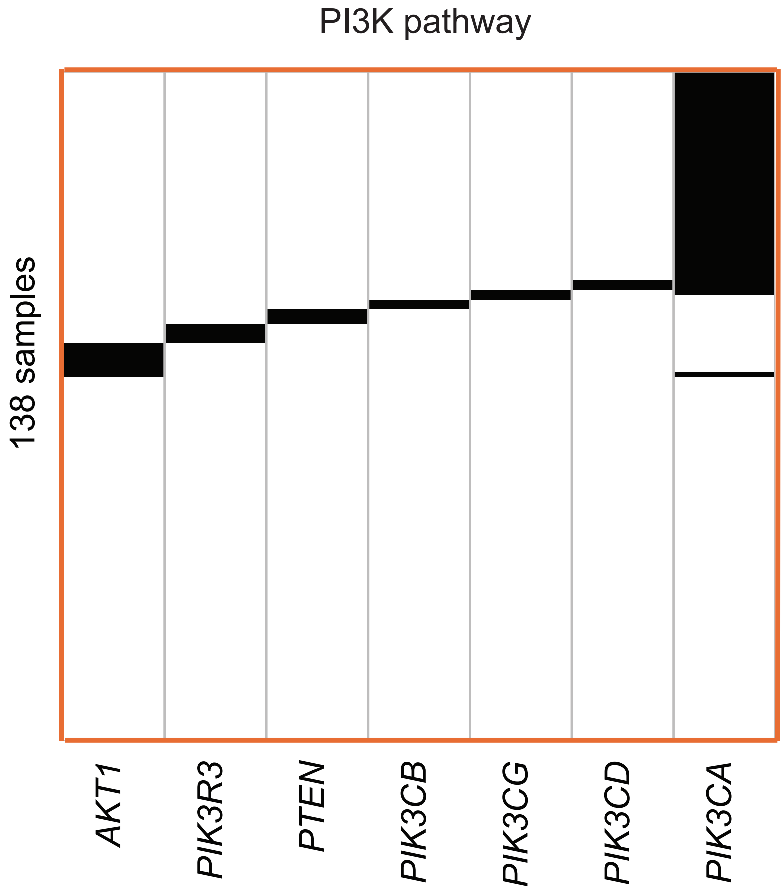

Fig S13. Recurrent CNAs segments

This figure shows (A) the average copy number profile and (B) the recurrently altered segments along the genome. Dashed lines indicate chromosome changes. The 165 recurrently altered segments were identified by ADMIRE[6].

The 1q gain is present in both subtypes, albeit at a lower level in the IR subtype. However, we clearly see the absence of the 8q gain in the IR subtype. The same holds for the 11q loss in the HR group. If the IR aberrations were of a similar magnitude as in HR, but detected at a lower level due to the cellularity difference, we would expect a smaller effect, not a complete absence of the 8q gain and 11q loss as we observe. Similarly the 6q loss is present in equal strength in both groups, and the IR group shows a loss of 18 not present in the HR group. Taken together, this shows no consistent modulation in copy number strength that could be ascribed to differences in cellularity that point towards a diminished power to detect aberrations in the IR group.

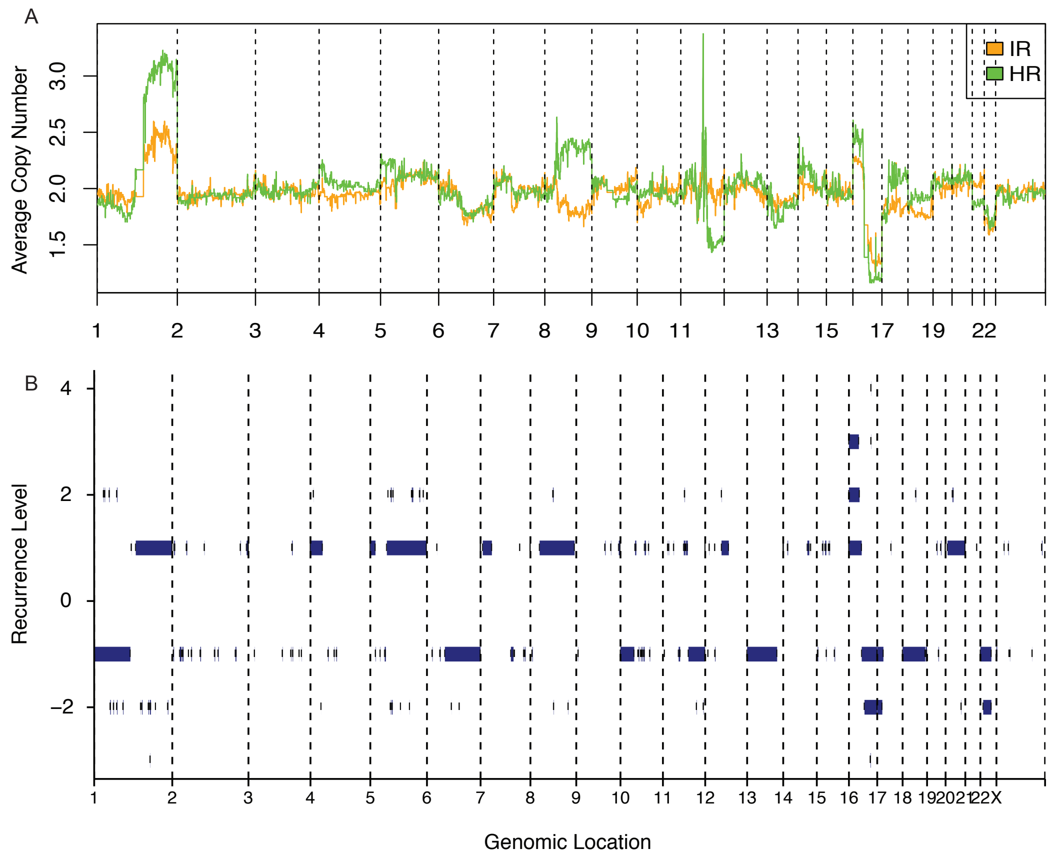

Fig S14. OncoScape candidate drivers

We show here the CNA, gene expression and RPPA values (log fold-change) in the IR and HR samples for the candidate drivers identified.

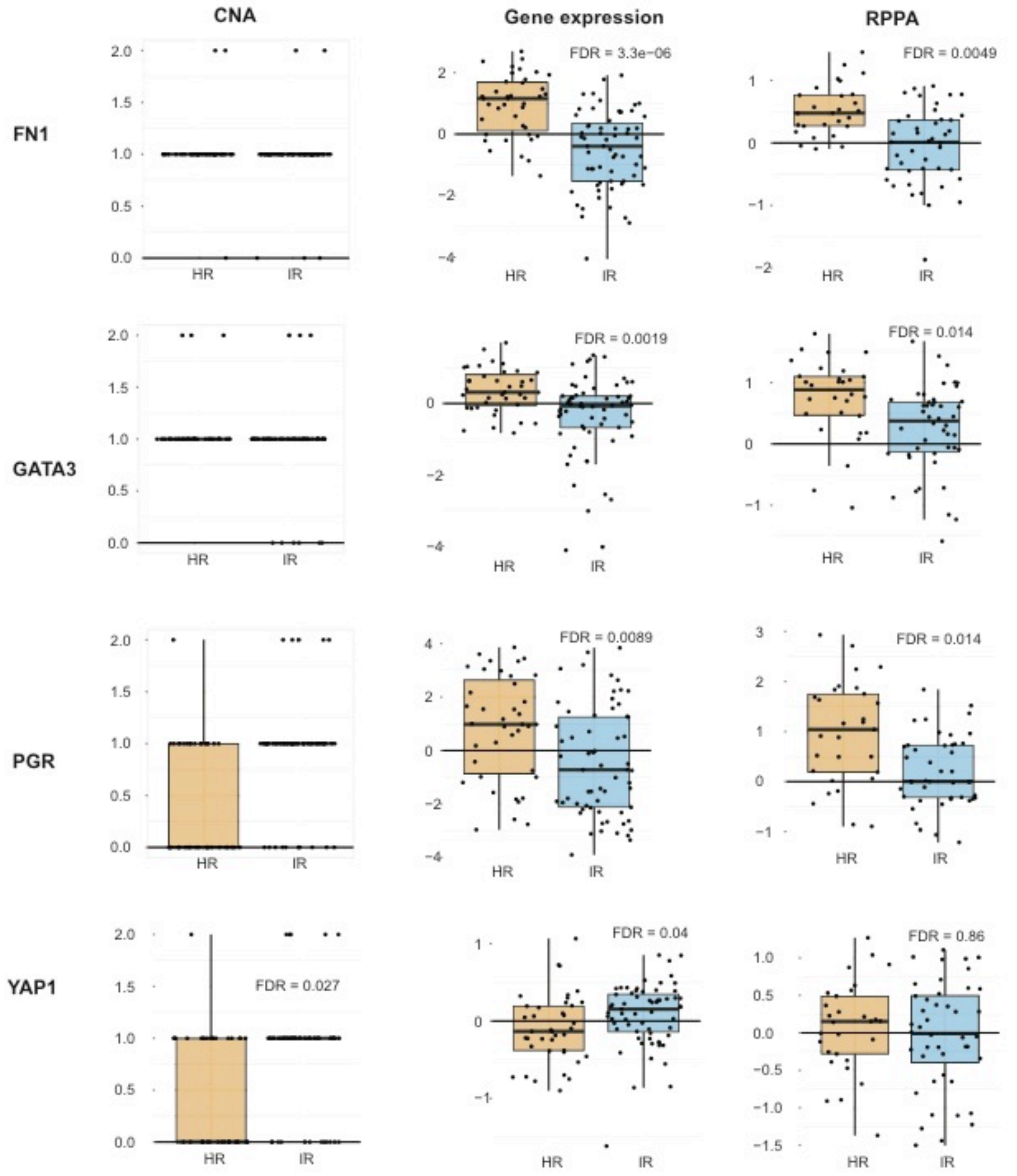

FIG S15. SURVIVAL ANALYSIS OF THE IR AND HR SUBTYPES

We show here the Kaplan-Meier plot of the stratification of the cohort based on the IR and HR subtypes. There is no significant difference in survival.

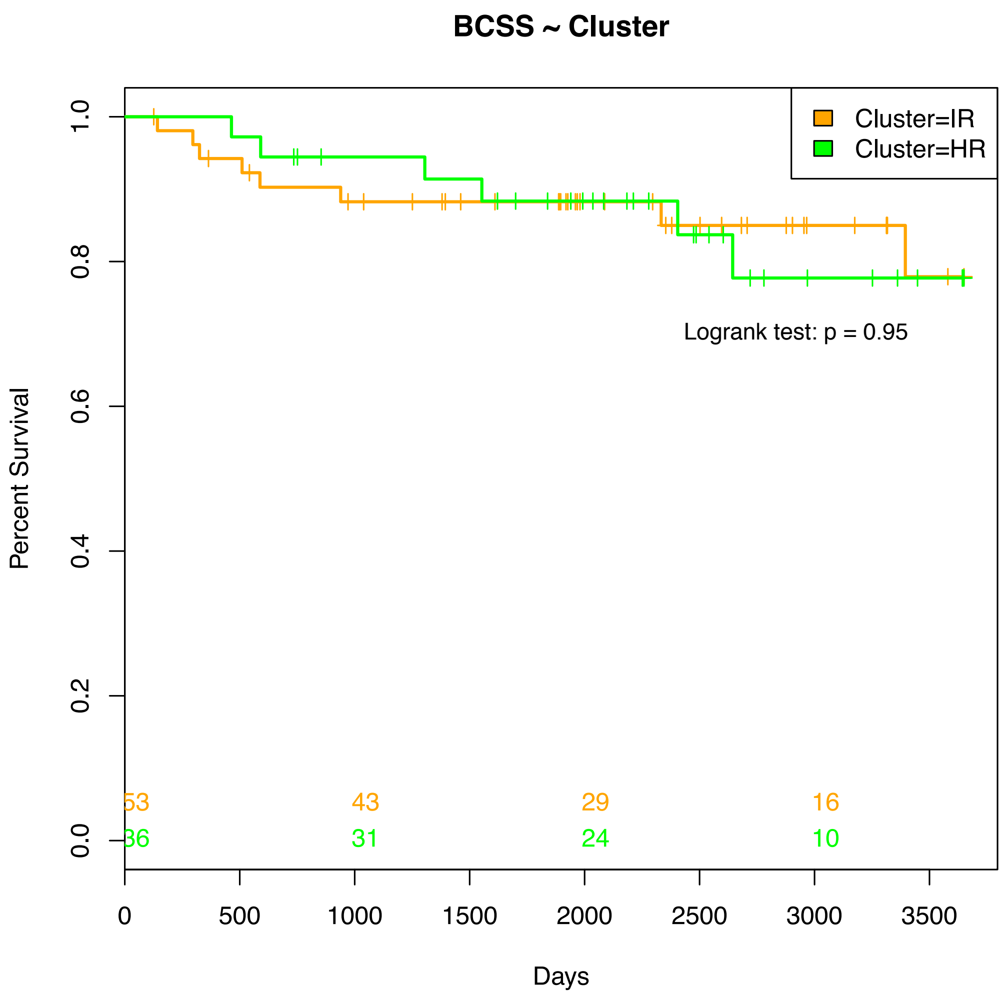

FIG S16. CELL LINES

15 ILC-like cell lines were selected as ILC-like based on genetic criterion. Using the gene expression data, we mapped them to both subtypes: 1) IR subtype: EVSAT, MPE600, HCC1187, MDAMB468, SkBr3, MDAMB453, OCUBF, OCUBM, SkBr5, HCC2218; 2) HR subtype: SUM44PE, MDAMB134VI, CAMA1, MDAMB330, ZR7530. 12 of these cell lines were screened for sensitivity to a large panel of drugs at the WTSI (EVSAT, HCC1187, MDAMB468, SkBr3, MDAMB453, OCUBF, OCUBM, HCC2218 and MDAMB134VI, CAMA1, MDAMB330, ZR7530).

Cell line	*E-cadherin*	*α -catenin*
CAMA-1	Splice site mutation c.1712-1G>A	
EVSA-T	Splice site mutation c.687+1delGT	
HCC1187		nonsense mutation c.2032C>T/p.Q678X
HCC2218	Large scale deletion c.1-832del	
MDA-MB-134-VI	Large scale deletion c.688-832del	
MDA-MB-330		nonsense mutation c.1322C>G/p.S441X
MDA-MB-453	nonsense mutation p.W638*	
MDA-MB-468		Large scale deletion c.302_588del287
MPE-600	Splice site mutation SA exon9 del	
OCUB-F	Large scale deletion	
OCUB-M	Other reason caused no protein expression	
SK-BR-3	Large scale deletion	
SK-BR-5	Splice site mutation SA exon5 ag>ac	
SUM-44-PE	Other reason caused no protein expression	
ZR-75-30	nonsense mutation p.E243*	

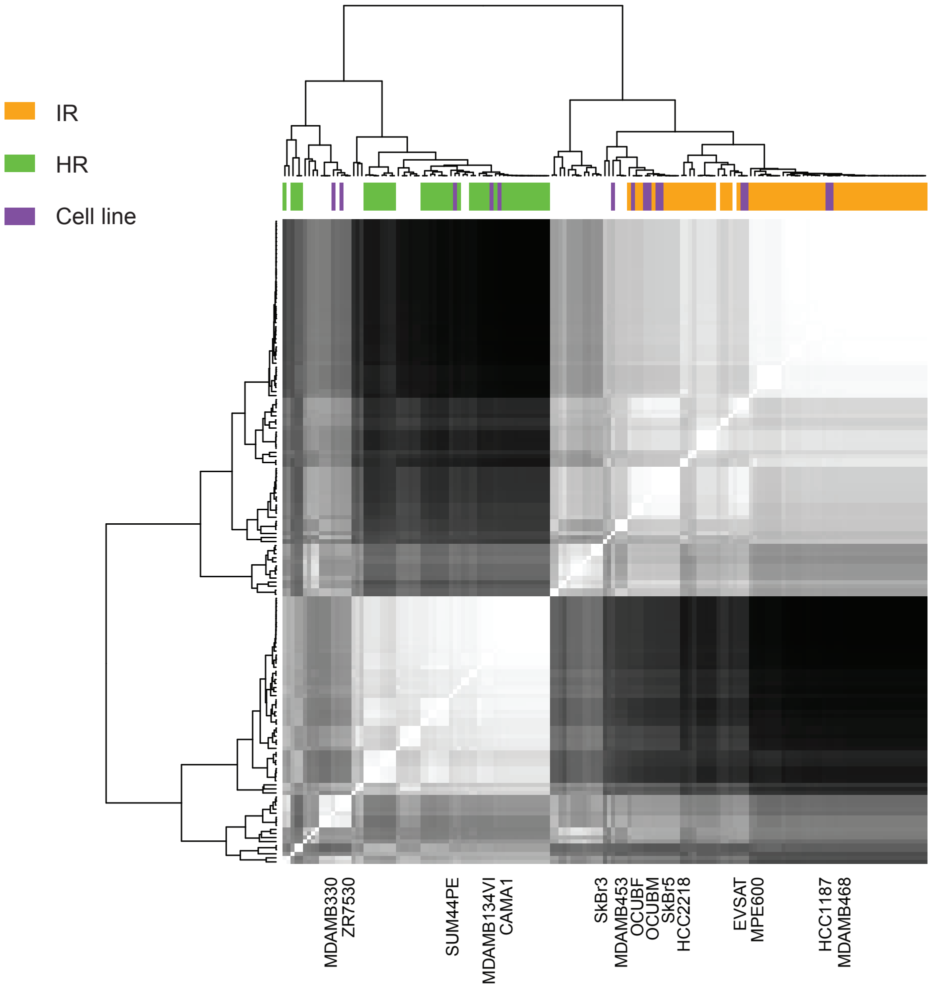

FIG S17. DIFFERENTIAL DRUG RESPONSE

The boxplots show the drug response (ln IC50) of the cell lines in the IR and HR subtypes for the six drugs with FDR<0.25. The red dotted line is the maximum screening concentration. The first three drugs in the figure are DNA-damaging agents.

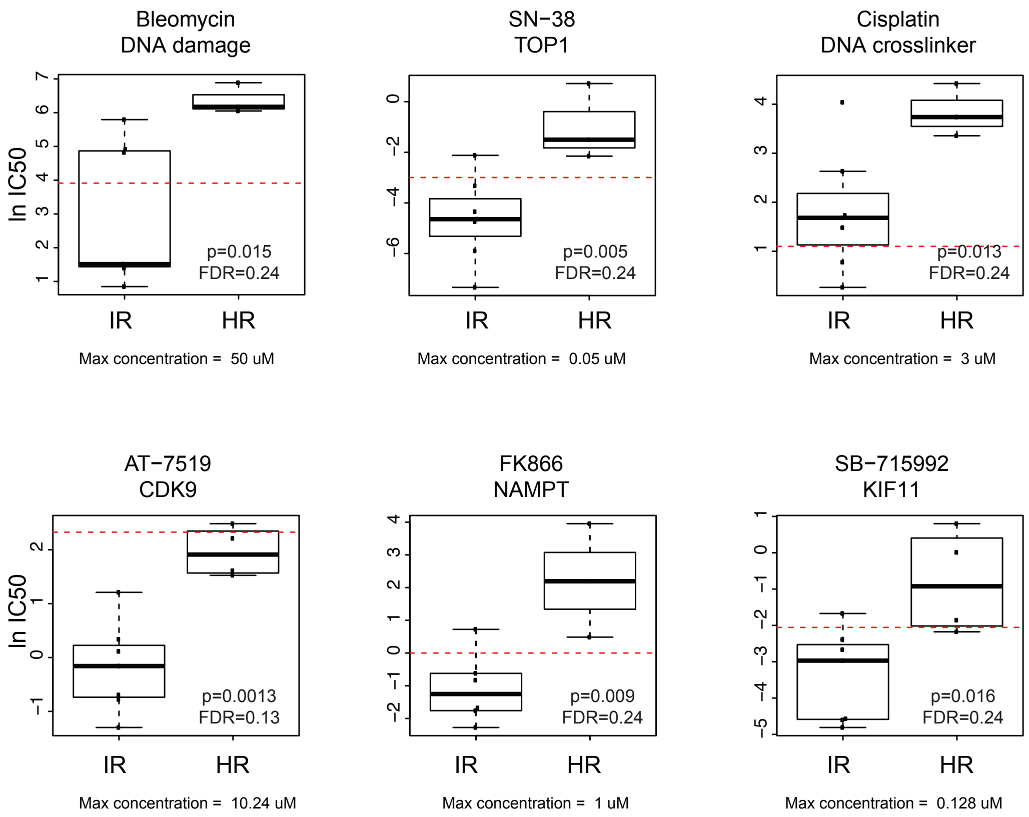

FIG S18. SURVIVAL ANALYSIS OF THE MUTATION RATE

We show here the Kaplan-Meier plot of the stratification of the cohort based on the somatic mutation rate. Patients which tumours have a high number of protein-altering somatic mutations (10 and more) have a poor survival.

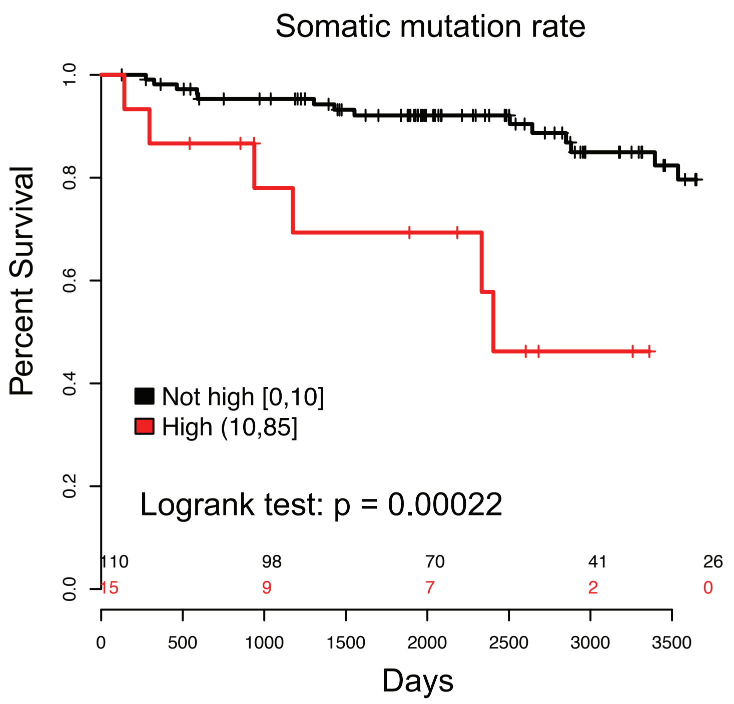

FIG S19. SURVIVAL ANALYSIS OF PROTEINS

A list of 18 proteins was found to be associated with survival (Additional file 11). In particular (A) higher level of eIF4B is associated with poor survival, while (B) higher level of histone H2AX is associated with better survival.

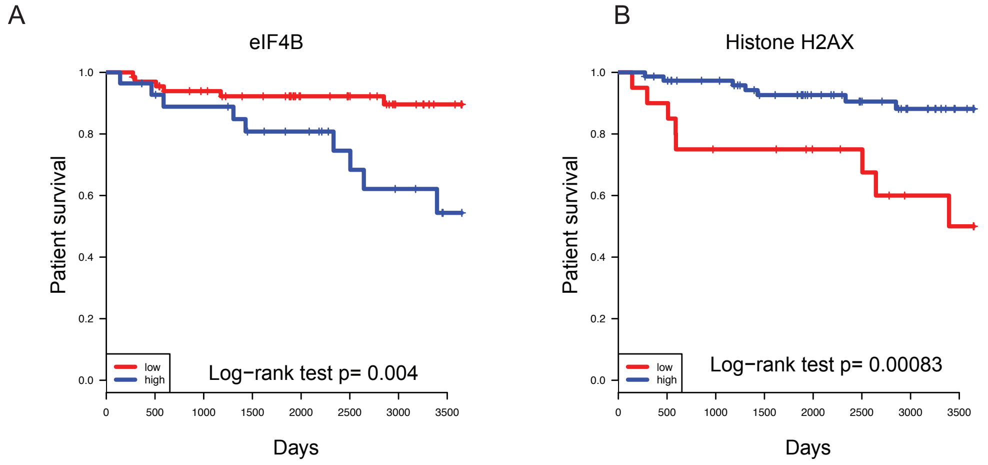

FIG S20. DECISION TREE PERFORMANCE ASSESSMENT

To assess the robustness of the result, we performed internal cross-validation of the different models and of the features as shown in the flow chart. We assessed the performance of different models with leave-one-out cross-validation (LOOCV). Each model is assessed with Cox regression. We illustrate in the chart how we computed the partial likelihood deviance. We show the results of the partial likelihood deviance for each model. We find that the selected features (mutation rate, eIF4B level) perform well, but the cluster assignments not, suggesting that while the cutoffs are specific to the dataset, the features are robust.

Performance assessment of the models

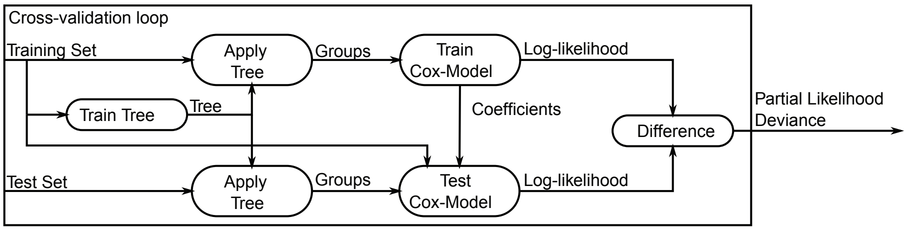

Performance assessment of the features

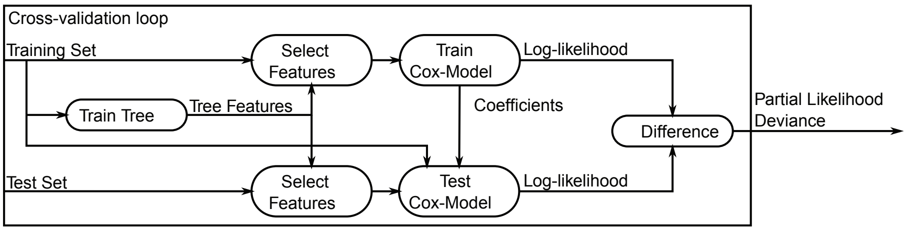

Performance by LOOCV of Cox models

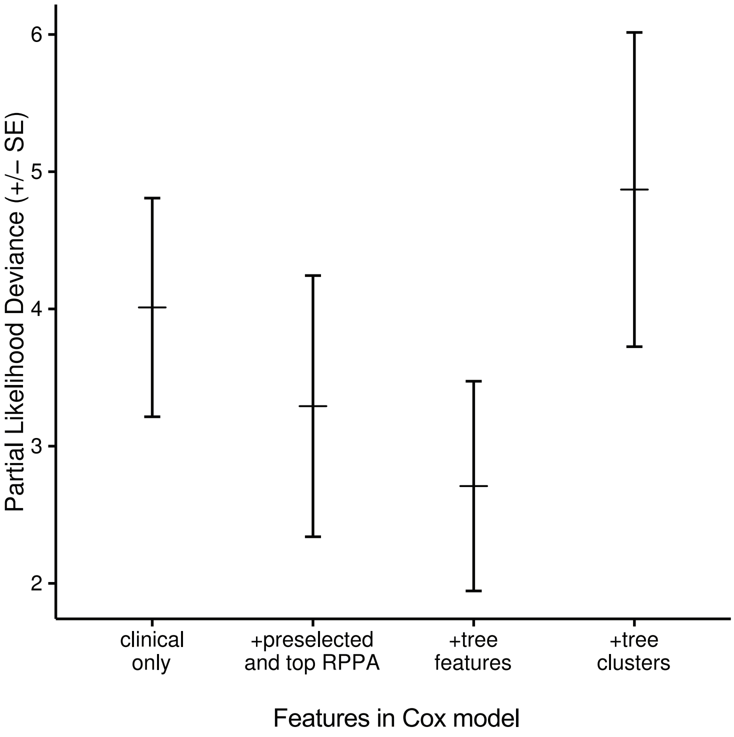

FIG S21. DECISION TREE WITH CLINICAL VARIABLES

When we add to the inputs the commonly used clinical variables, we find a similar tree with only lymph node status as an additional tree node. This results in the selection of a few patients with a very high number of positive lymph nodes (>6) and a very poor survival. The good prognosis group (eIF4B low) becomes even better with only two events.

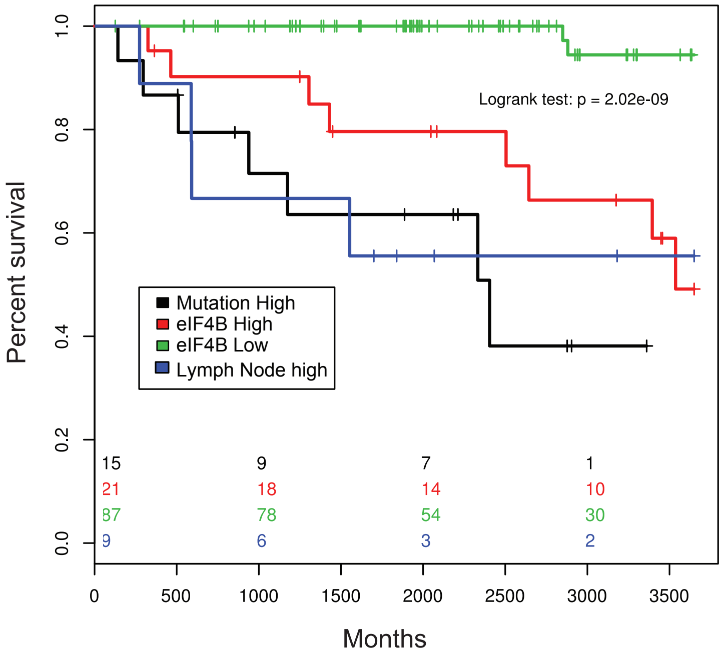

FIG S22. POSSIBLE TREATMENT EFFECT

Possible treatment effect indicated by decision trees in treated / untreated groups. Decision trees obtained for patients without hormonal treatment (A) and patients with hormonal treatment (B). The samples with low mutation rate and high eIF4B show poor survival without hormonal treatment, but good survival with hormonal treatment. We could not test this in a Cox model, because of the limited number of samples.

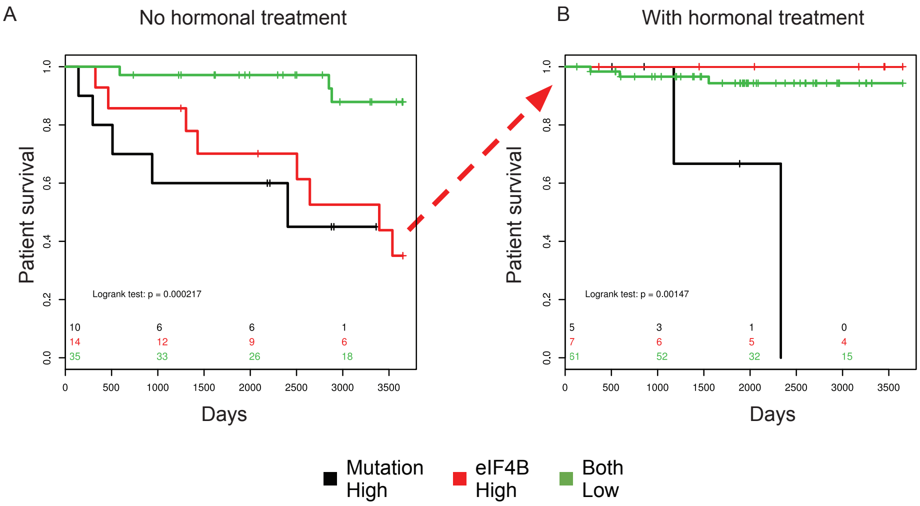

FIG S23. SUBTYPE BIOMARKERS AND LYMPHOCYTIC INFILTRATION

To investigate whether the tumours with high immune gene expression (as represented by CD8A expression) show low levels of GATA3, ESR1 and PGR, we scattered each of these proteins against CD8A RNAseq counts. The plot shows the CD8A gene expression as FPKM and protein expression of A) PGR; B) GATA3; C) ESR1 and D) phosphorylation of ESR1 on Ser118. Samples from IR/HR/unassigned are represented in orange/green/black. Orange, green and black dots represent the IR, HR and unassigned samples. If high immune response were associated with low expression of these proteins we would expect a negative correlation in these plots. This is not the case. In fact, we observe no (anti-)correlation at all, but observe both high and low protein expression at both high as well as low CD8A mRNA expression levels. This is the case for PGR (Panel A), GATA3 (Panel B), ESR1 (Panel C) as well as the phosphorylation level of Ser118 on ESR1 (Panel D). In summary, while CD8A, ESR1, PGR and GATA3 are all individually associated with the subtypes, this association does not arise due to the different levels of immune cells in the subtypes, as all four proteins show no association with CD8A (as marker for immune cells).

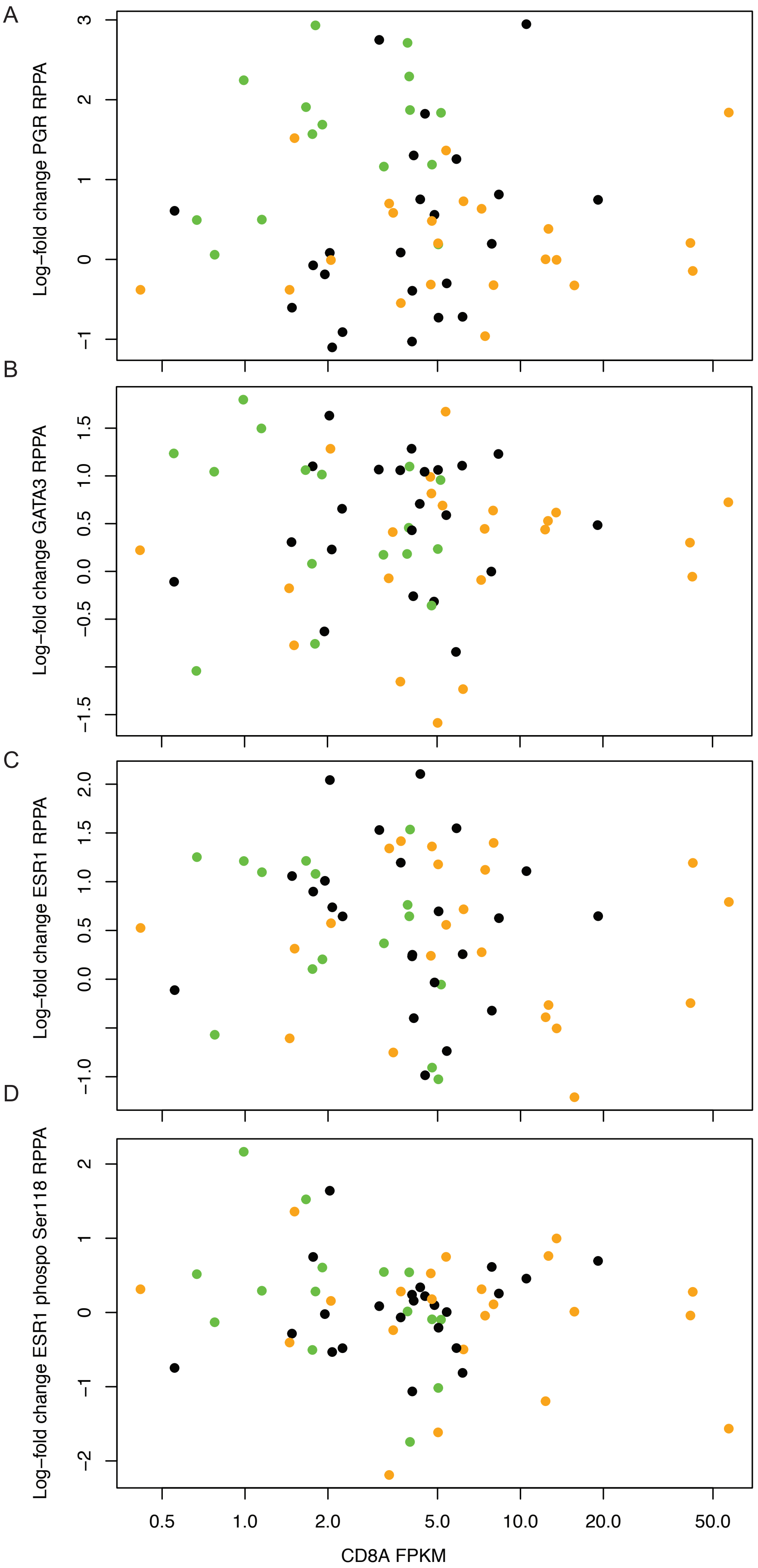
A
B
C
D
Log−fold change PGR RPPA
Log−fold change GATA3 RPPA
Log−fold change ESR1 RPPA
Log−fold change ESR1 phospo Ser118 RPPA
−1
0
1
2
3
−1.5
−0.5
0.0
0.5
1.0
1.5
−1.0
0.0
0.5
1.0
1.5
2.0
−2
−1
0
1
2
0.5
1.0
2.0
5.0
10.0
20.0
50.0
CD8A FPKM

Fig S24. PD-L1 immunohistochemitry

We have performed IHC staining and scoring of 29 of our samples with lymphocytic infiltration. Four of the samples show some PD-L1 staining. Even if it represents less than 1% of the cells, we note that this staining is in both immune and tumour cells.

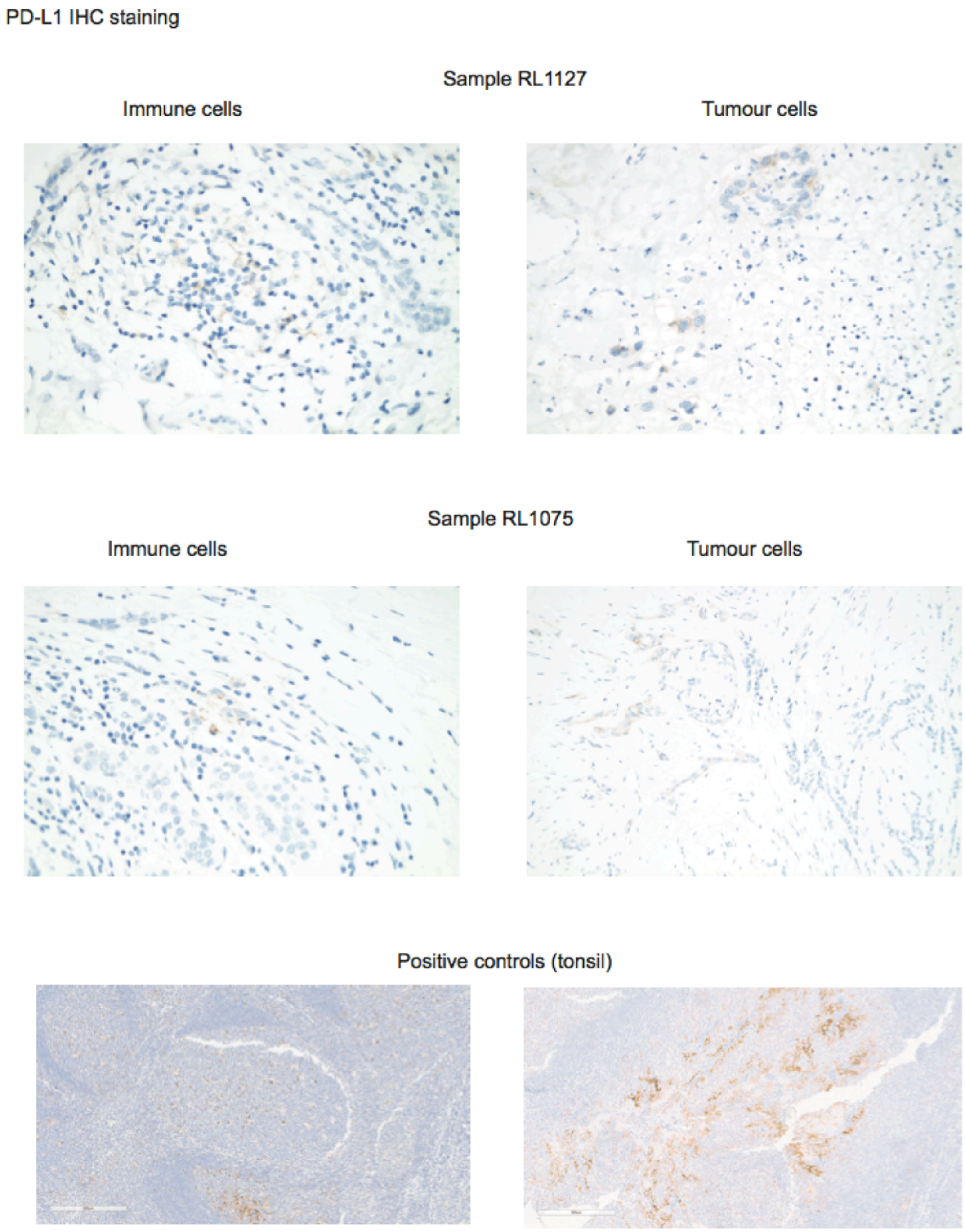

Fig S25. RPPA clustering

We show here the heatmap of the RPPA data with sample and epitope names.

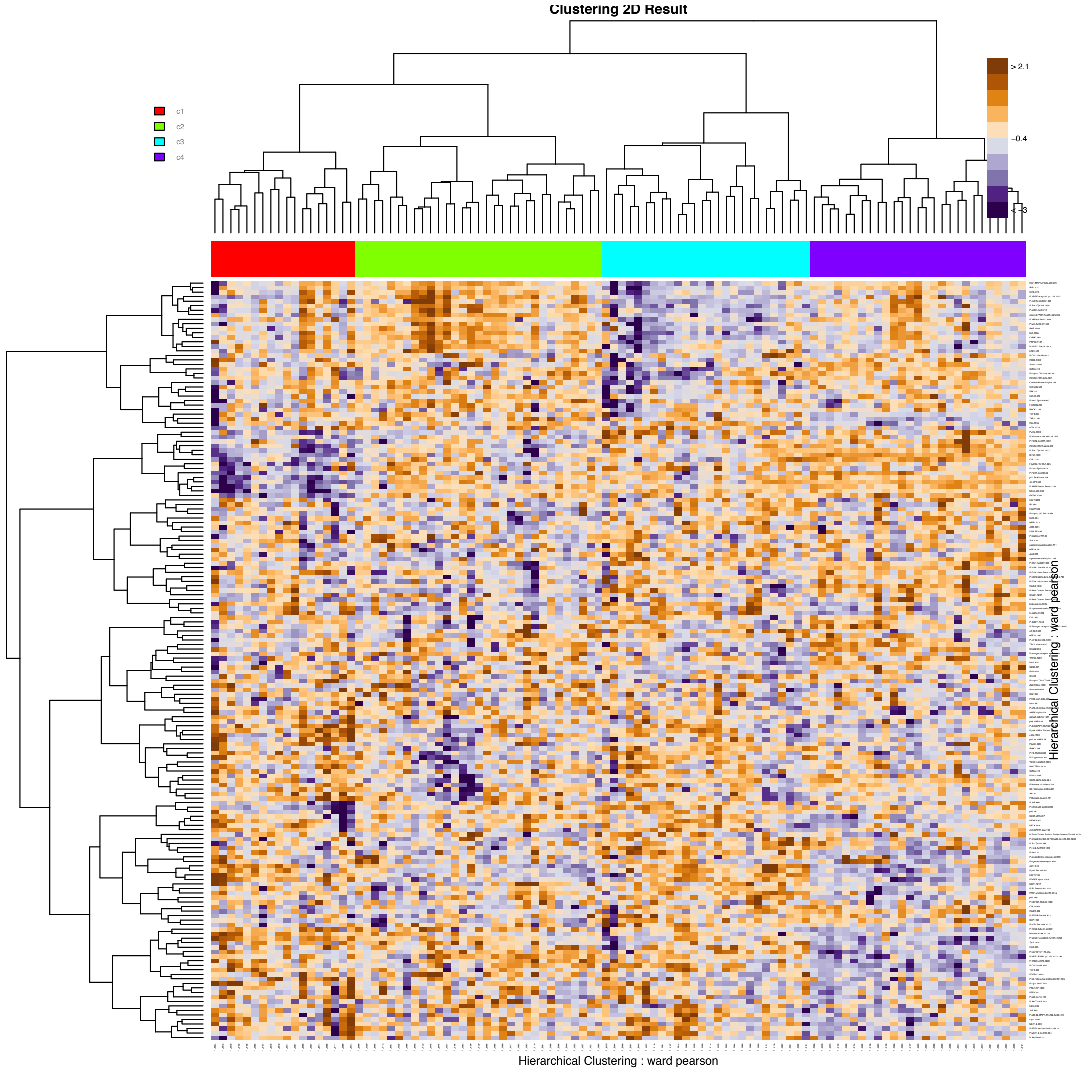

Supplementary Tables

Table S1. Integrative clusters on RATHER

The RATHER samples were classified into the 10 integrative clusters from METABRIC using the iC10 package with default parameters and the "scale" normalization method[5]. We report here the IR and HR subtypes relation to these 10 integrative clusters. Using an Asymptotic Pearson's Chi-Squared Test, we find that both subtyping results are associated (p<5e-4).

	iC1	iC2	iC3	iC4	iC5	iC6	iC7	iC8	iC9	iC10
IR	1	0	**25**	**16**	1	0	2	**12**	0	0

HR	1	4	**12**	0	1	1	6	**10**	3	0

Table S2. Lymphocytic infiltration

We scored the lymphocytic infiltration on the RATHER samples in the IR and HR subtypes. The scoring system was as follows: MILD when lymphocytes are scattered, discrete; INTERMEDIATE when lymphocytes are scattered, some areas are dense with lymphocytes; SEVERE when lymphocytes are confluent sheets of cells. 13 slides were of poor quality and could not be scored (NA).

	MILD	INTERMEDIATE	SEVERE	NA
HR	17	11	10	1
IR	11	19	21	12

Table S3. IR/HR and TCGA subtypes

We report here the overlap between the IR/HR subtypes defined in that work and the subtypes defined by TCGA on A) TCGA samples and B) METABRIC samples. On TCGA samples, the Reactive-like subtype is associated with the IR subtype, while Immune-related and Proliferative subtypes are associated with the HR subtype (Chi-squared p-value<1e-6). On the METABRIC samples, the subtypes do not show association (Chi-squared p-value=0.47).

A: on 115 TCGA samples	Immune-related	Proliferative	Reactive-like
IR	7	6	37
HR	36	19	4
NA	3	0	3

B: on 103 METABRIC samples	Immune-related	Proliferative	Reactive-like
IR	19	13	17
HR	14	21	17
NA	1	1	0

Table S4. Intrinsic and integrative clusters on METABRIC

We report here the IR and HR subtypes relation to (A) the intrinsic subtypes of the 103 samples of the METABRIC validation set as defined in the original study (one sample was not classified – NC); and on the 10 METABRIC integrative clusters considering (B) all the 103 samples, (B) the 57 samples not luminal A and (C) the 46 luminal A samples. We find that the luminal A samples are equally distributed between IR and HR subtypes. Luminal B is associated with HR, while normal-like is associated with IR. IntClust 4, characterized by lymphocytic infiltration, is almost exclusively in IR, but also mostly in non Luminal A samples. (E) When clustering only the luminal A samples, we can recover the IR and HR subtypes. Thus, IR and HR are distinct subtypes found in luminal A ILC samples, with some normal-like component in IR and some luminal B component in HR.

A. Intrinsic subtypes in all samples (Chi-squared p=5e-5)

	Basal	Her2	LumA	LumB	NC	Normal
**HR**	0	3	*21*	*23*	1	4
**IR**	4	3	*23*	1	0	*18*
**NA**	0	0	2	0	0	0

B. Integrative clusters on all samples

	iC1	iC2	iC3	iC4	iC5	iC6	iC7	iC8	iC9	iC10
**HR**	3	1	*15*	1	0	2	4	*22*	3	1
**IR**	0	1	*21*	*21*	1	0	1	2	0	2
**NA**	0	2	0	0	0	0	0	0	0	0

C. Integrative clusters on non luminal A samples

	iC1	iC2	iC3	iC4	iC5	iC6	iC7	iC8	iC9	iC10
**HR**	3	1	*6*	0	0	1	2	*15*	3	0
**IR**	0	0	*6*	*17*	1	0	0	0	0	2

D. Integrative clusters on Luminal A samples

	iC1	iC2	iC3	iC4	iC5	iC6	iC7	iC8	iC9	iC10
**HR**	0	0	*9*	1	0	1	2	*7*	0	1
**IR**	0	1	*15*	4	0	0	1	2	0	0
**NA**	0	2	0	0	0	0	0	0	0	0

E. Clustering the luminal A samples

METABRIC	IR-LumA	HR-LumA	NA-LumA
IR	18	0	5
HR	1	15	5
NA	0	0	2

Table S5. Systematically comparing IR and HR subtypes

The table indicates the odds ratio of the presence of a given feature in the IR versus HR subtypes as shown in Figure 1, and the 95% confidence intervals (CI). We performed a Fisher's exact test and corrected the p-values with the Benjamini-Hochberg correction of multiple testing.

**Feature**	**Odds Ratio**	**CI low**	**CI high**	**P-value**	**FDR**
CDH1	2.1	0.141	30.9	0.5948	0.811
PIK3CA	1.3	0.497	3.3	0.6589	0.811
PI3K	1.5	0.615	3.8	0.4008	0.641

GATA3	0	0	3.5	0.2697	0.594
ERBB2	6.6	0.618	335.3	0.0783	0.25
NF1	1.5	0.019	122	1	1
MAP3K1	4.8	0.369	260.5	0.2972	0.594
MAP2K4	0	0	2.3	0.1499	0.4
TP53	1.5	0.019	120.8	1	1
High mutation rate	1.3	0.428	4	0.614	0.811
1q gain	3.3	1.261	9.2	0.0102	0.082
8q gain	3.3	1.05	11.3	0.0337	0.18
11q loss	4.3	1.549	12.5	0.0024	0.039
ER	Inf	0.656	Inf	0.0753	0.25
PR	1.7	0.61	5	0.3498	0.622
HER2	1.5	0.102	21.1	1	1

TABLE S6. ADJUSTED HAZARD RATIOS FOR KNOWN PROGNOSTIC FACTORS

We report here the adjusted hazard ratios (HR) for the commonly used clinical variables, with 95% confidence intervals (CI).

Coefficient	HR	CI low	CI high
tumour_size_in_cm	1.004	0.8428	1.196
histological_grade	0.5671	0.06455	4.983
number_of_positive_lymph_nodes	1.31	1.143	1.501
treatment_hormonalTRUE	0.1266	0.02253	0.7119
treatment_radiotherapyTRUE	0.2665	0.05138	1.382
treatment_adjuvant_chemotherapyTRUE	0.6387	0.09494	4.297
age_at_diagnosis	1.097	1.037	1.161

TABLE S7. HIGH CELLULARITY CLUSTERING

We have performed a separate clustering for samples with at least 50% tumor cellularity and report here the assignment of these samples (IR-50, HR50) with respect to the previously defined IR and HR subtypes. Only 3 samples are misclassified.

	IR	HR
IR-50	13	0
HR-50	3	26

REFERENCES

1 Anastassiou, D. *et al.* Human cancer cells express Slug-based epithelial-mesenchymal transition gene expression signature obtained in vivo. *BMC cancer* **11**, 529, doi:10.1186/1471-2407-11-529 (2011).

2 Curtis, C. *et al.* The genomic and transcriptomic architecture of 2,000 breast tumours reveals novel subgroups. *Nature* **486**, 346-352, doi:10.1038/nature10983 (2012).

3 Wang, K. *et al.* PennCNV: an integrated hidden Markov model designed for high-resolution copy number variation detection in whole-genome SNP genotyping data. *Genome research* **17**, 1665-1674, doi:10.1101/gr.6861907 (2007).

4 Van Loo, P. *et al.* Allele-specific copy number analysis of tumors. *Proc Natl Acad Sci U S A* **107**, 16910-16915, doi:10.1073/pnas.1009843107 (2010).

5 Ali, H. *et al.* Genome-driven integrated classification of breast cancer validated in over 7,500 samples. *Genome biology* **15**, 431, doi:10.1093/jnci/dju049 (2014).

6 van Dyk, E., Reinders, M. J. T. & Wessels, L. F. A. A scale-space method for detecting recurrent DNA copy number changes with analytical false discovery rate control. *Nucleic acids research*, doi:10.1093/nar/gkt155 (2013).

7 Ross, J. S. *et al.* Relapsed classic E-cadherin (CDH1)-mutated invasive lobular breast cancer shows a high frequency of HER2 (ERBB2) gene mutations. *Clinical cancer research : an official journal of the American Association for Cancer Research* **19**, 2668-2676, doi:10.1158/1078-0432.CCR-13-0295 (2013).

8 Kim, D. *et al.* TopHat2: accurate alignment of transcriptomes in the presence of insertions, deletions and gene fusions. *Genome biology* **14**, R36, doi:10.1186/gb-2013-14-4-r36 (2013).

9 Liao, Y., Smyth, G. K. & Shi, W. featureCounts: an efficient general purpose program for assigning sequence reads to genomic features. *Bioinformatics (Oxford, England)* **30**, 923-930, doi:10.1093/bioinformatics/btt656 (2014).

10 Love, M. I., Huber, W. & Anders, S. Moderated estimation of fold change and dispersion for RNA-seq data with DESeq2. *Genome biology* **15**, 550, doi:10.1186/s13059-014-0550-8 (2014).

11 Wilkerson, M. D. & Hayes, D. N. ConsensusClusterPlus: a class discovery tool with confidence assessments and item tracking. *Bioinformatics (Oxford, England)* **26**, 1572-1573, doi:10.1093/bioinformatics/btq170 (2010).

12 Delahaye, L. J. *et al.* Performance characteristics of the MammaPrint® breast cancer diagnostic gene signature. *Personalized Medicine* **10**, 801-811, doi:10.2217/pme.13.88 (2013).

13 Bolstad, B. M., Irizarry, R. A., Astrand, M. & Speed, T. P. A comparison of normalization methods for high density oligonucleotide array data based on variance and bias. *Bioinformatics (Oxford, England)* **19**, 185-193 (2003).

14 Johnson, W. E., Li, C. & Rabinovic, A. Adjusting batch effects in microarray expression data using empirical Bayes methods. *Biostatistics (Oxford, England)* **8**, 118-127, doi:10.1093/biostatistics/kxj037 (2007).

15 Troncale, S. *et al.* NormaCurve: A SuperCurve-Based Method That Simultaneously Quantifies and Normalizes Reverse Phase Protein Array Data. *PloS one* **7**, e38686, doi:10.1371/journal.pone.0038686 (2012).

16 Vogelstein, B. *et al.* Cancer genome landscapes. *Science (New York, N.Y.)* **339**, 1546-1558, doi:10.1126/science.1235122 (2013).

17 Shen, R., Olshen, A. B. & Ladanyi, M. Integrative clustering of multiple genomic data types using a joint latent variable model with application to breast and lung cancer subtype analysis. *Bioinformatics (Oxford, England)* **25**, 2906-2912, doi:10.1093/bioinformatics/btp543 (2009).

18 Subramanian, A. *et al.* Gene set enrichment analysis: a knowledge-based approach for interpreting genome-wide expression profiles. *Proceedings of the National Academy of Sciences of the United States of America* **102**, 15545-15550, doi:10.1073/pnas.0506580102 (2005).

19 Zwart, W. *et al.* Oestrogen receptor-co-factor-chromatin specificity in the transcriptional regulation of breast cancer. *The EMBO journal* **30**, 4764-4776, doi:10.1038/emboj.2011.368 (2011).

20 Hothorn, T. & Lausen, B. Bagging tree classifiers for laser scanning images: a data- and simulation-based strategy. *Artificial intelligence in medicine* **27**, 65-79 (2003).

21 Barbosa-Morais, N. L. *et al.* A re-annotation pipeline for Illumina BeadArrays: improving the interpretation of gene expression data. *Nucleic acids research* **38**, e17, doi:10.1093/nar/gkp942 (2010).
